# Duration-dependent effects of water-only fasting on blood lipids: a systematic review, meta-analysis, and threshold meta-regression

**DOI:** 10.3389/fnut.2026.1772246

**Published:** 2026-04-01

**Authors:** Ayşe Çamli, İzzet Ülker, Merve Terzi, Edanur Yağan, Serhat Özbay, Süleyman Ulupınar

**Affiliations:** 1 Department of Nutrition and Dietetics, Faculty of Health Sciences, Erzurum Technical University, Erzurum, Türkiye; 2 Department of Nutrition and Dietetics, Faculty of Health Sciences, Istanbul Yeni Yuzyil University, Istanbul, Türkiye; 3 Faculty of Sports Sciences, Erzurum Technical University, Erzurum, Türkiye

**Keywords:** blood lipids, HDL cholesterol, LDL cholesterol, meta-analysis, total cholesterol, triglycerides, VLDL cholesterol, water-only fasting

## Abstract

**Background/objectives:**

Water-only fasting is practiced for metabolic and therapeutic purposes, yet its specific effects on lipid fractions remain inconsistently reported. This systematic review and meta-analysis evaluated lipid responses to water-only fasting across varying durations and fasting protocols.

**Methods:**

PubMed, Scopus, and Web of Science (2000–2025) were searched for human studies reporting pre–post lipid measurements under water-only fasting. Thirty-two studies met eligibility criteria. Effect sizes were calculated as Hedges’ g using random-effects models. Duration-dependent responses were evaluated through subgroup analyses (≤3 days, >3 days) and piecewise threshold meta-regression. Publication bias was assessed via funnel plots and Egger’s tests.

**Results:**

Water-only fasting produced lipid-specific and duration-dependent adaptations. HDL decreased significantly overall (g = −0.233), with no change in ≤3-day fasts but clear reductions in >3-day fasts; threshold analysis identified an early decline within the first ~3 days. LDL increased significantly (g = 0.489) and across all duration subgroups, showing a biphasic trajectory with progressive elevation up to ~10 days followed by attenuation or partial reversal. Total cholesterol also increased (g = 0.343), with the largest effects in >3–-day fasts and a nonlinear threshold at ~5 days marking stabilization or modest decline thereafter. Triglycerides showed no significant overall effect (g = −0.039), characterized by reductions in ≤3-day fasts, increases in >3-day fasts; a marked early-phase threshold was observed at ~2.5 days. VLDL exhibited small, non-significant changes (g = 0.203) with substantial heterogeneity and limited data. Evidence of publication bias was detected for LDL and total cholesterol but not for HDL, triglycerides, or VLDL.

**Conclusion:**

Water-only fasting induces distinct, duration-dependent lipid adaptations. LDL and total cholesterol demonstrate early increases followed by stabilization, HDL decreases mainly during multi-day fasts, while triglycerides and VLDL show no uniform pattern. These findings highlight the importance of considering fasting duration when evaluating cardiometabolic effects and underscore the need for rigorously controlled, longer-term clinical trials.

## 1 Introduction

Dyslipidemia, characterized by abnormal blood lipid concentrations, represents a major modifiable risk factor for cardiovascular diseases, the leading cause of death worldwide (1). Traditional management strategies typically include pharmacological interventions and dietary modifications aimed at increasing high-density lipoprotein cholesterol (HDL-C) while reducing total cholesterol (TC), low-density lipoprotein cholesterol (LDL-C), very low-density lipoprotein cholesterol (VLDL-C), and triglycerides (TG) (2). However, the metabolic complexity of lipid regulation and individual variability in response to therapy have led to the exploration of alternative treatment approaches. Fasting, a practice that has been practiced in various cultures and religions for thousands of years, has the potential to positively affect cardiometabolic health markers, making it an interesting area of research in preventive and therapeutic medicine (3–8).

Water-only fasting (WOF) is a powerful metabolic intervention where only water is allowed and calorie intake is completely cut off (9). The profound impact of water-only fasting on lipid profiles begins with a fundamental metabolic shift. In the fed state, the human body primarily utilizes glucose for energy production, with lipid metabolism playing a secondary role. However, within 12–24 h of complete food abstinence, hepatic glycogen stores are depleted, necessitating a switch to alternative fuel sources. This metabolic reprogramming triggers accelerated lipolysis in adipose tissue, leading to the release of free fatty acids (FFAs) into the circulation, which are then utilized by peripheral tissues for oxidation and conversion to ketone bodies in the liver (10). This dramatic increase in lipid mobilization and oxidation provides the mechanistic basis for the effects of fasting on circulating lipid concentrations; however, the relationship between fasting and blood lipid levels reveals a complex and sometimes paradoxical pattern (3, 6, 7). One of the most striking and clinically important findings in water-only fasting studies is the acute elevation in lipid parameters during the fasting period (11, 12). Numerous studies have documented that fasting causes significant increases in total cholesterol (5, 11, 13–19), LDL-C (5, 11, 16–21), and triglycerides (7, 12, 14, 16, 17, 19, 22, 23). Studies examining 10-day water-only fasting protocols have revealed that total cholesterol levels increase significantly during the fasting period (5, 15, 19). Similarly, LDL-C generally rises after short-term fasting (11, 20, 21), and studies have documented increases that would be considered clinically significant if sustained over the long term (5, 16, 18). Although triglyceride levels have been reported to increase significantly, some studies have also seen decreases (8, 11, 18, 24–27) or stability (28). HDL-C levels are generally reported to decrease with fasting (7, 15, 17, 19, 28, 29), while inconsistent changes are observed, with studies reporting both increases (7, 12) and decreases (30–32) in VLDL-C levels. Adverse changes, such as increases in lipid levels, observed during water-only fasting, suggest that this fast may not be suitable for patients with dyslipidemia and requires close monitoring of lipid levels (17).

While numerous studies have evaluated the metabolic effects of water fasting alone, the evidence is inconsistent regarding changes in blood lipid levels, largely due to heterogeneity in fasting duration, participant characteristics, and study design. To our knowledge, no studies to date have systematically examined the relationship between fasting duration and changes in blood lipid parameters. Conflicting findings in the literature suggest that the effect of water-only fasting on lipid profiles may not follow a linear pattern but instead exhibit a behavior dependent on the duration of fasting. Therefore, this systematic review and meta-analysis study aims to systematically examine the effects of water-only fasting on blood HDL-C, LDL-C, VLDL-C, triglyceride and total cholesterol levels to resolve inconsistencies in the existing literature, to analyze changes depending on fasting duration using threshold meta-regression method and to determine clinically significant duration thresholds in terms of lipid profile.

## 2 Methods

### 2.1 Literature search strategy

A comprehensive literature search was performed in PubMed, Scopus, and Web of Science (WoS) to identify studies examining the effects of water-only fasting on serum lipid parameters in humans. The search covered publications from January 2000 to October 30, 2025, limited to peer-reviewed articles in English involving human participants.

The search strategy employed Boolean operators to combine fasting-related and lipid-related keywords (e.g., “water-only fasting,” “prolonged fasting,” “lipid profile,” “cholesterol,” “triglycerides”). Each database query was specifically adapted to its indexing system to maximize sensitivity and specificity.

In total, 414 records were identified (PubMed = 137, Scopus = 120, WoS = 157). After removing duplicates, 258 unique studies were screened for eligibility. The reference lists of included papers and relevant reviews were also hand-searched to capture any additional eligible studies.

The detailed Boolean search strings for each database are provided in the Supplementary Table S1. The search and selection process followed the PRISMA 2020 guidelines, and a PRISMA flow diagram illustrating study identification, screening, and inclusion was prepared accordingly.

### 2.2 Eligibility criteria

Studies were included if they met all of the following criteria:

- *Population:* Human participants aged ≥18 years, regardless of sex, ethnicity, or baseline health status.
- *Intervention:* Water-only fasting (i.e., complete abstinence from food and caloric beverages), with or without water mineralization, lasting for at least 24 h.
- *Study design:* Experimental, quasi-experimental, or longitudinal pre–post studies reporting baseline and post-fasting measurements.
- *Outcomes:* At least one lipid parameter reported before and after fasting, including total cholesterol (TC), high-density lipoprotein (HDL), low-density lipoprotein (LDL), very-low-density lipoprotein (VLDL), or triglycerides (TG).
- *Data availability:* Studies that presented sufficient data for effect size calculation (mean and standard deviation), or data convertible from standard error (SE), median and interquartile range (IQR), or graphical formats (digitized using WebPlotDigitizer, v4.x).
- *Publication characteristics:* Full-text, peer-reviewed articles published in English between January 2000 and October 30, 2025.

Studies were excluded if they met any of the following criteria:

- Animal or cell-based experiments.
- Fasting protocols that included caloric intake, supplements, or non-water beverages (e.g., juice, tea, or coffee).
- Religious fasting practices (e.g., Ramadan fasting) or intermittent fasting schedules (e.g., 16:8, alternate-day fasting).
- Studies focusing exclusively on disease-specific populations (e.g., diabetes, cardiovascular disease) without a healthy or mixed control group.
- Review articles, meta-analyses, conference abstracts, case reports, or editorials lacking primary data.

### 2.3 Data extraction and transformation

Two independent reviewers (AÇ and SU) extracted the data from all eligible studies using a standardized extraction form. The extracted information included study identifiers (author, year, country), participant characteristics (sample size, sex, age, fasting duration), study design, fasting protocol details, and lipid outcome measures. Any discrepancies between reviewers were resolved through discussion and consensus.

Whenever available, the mean and standard deviation (SD) of pre- and post-fasting lipid parameters were recorded directly. However, since data reporting formats varied considerably among studies, several transformation procedures were applied to ensure statistical comparability:

*Standard error (SE) to standard deviation (SD):* when results were expressed as mean ± SE, the SD was computed using:


$$\mathrm{SD}=\mathrm{SE}\times \sqrt{n}$$

*Median and interquartile range (IQR) to mean and SD:* For studies reporting medians and IQRs, the mean and SD were estimated using established transformation equations proposed by Hozo et al. (33) and refined by Wan et al. (34). These formulas were selected based on sample size and distributional assumptions.

*Graphically reported data:* For studies presenting data only in graphical form (e.g., bar or line charts), quantitative values were extracted using WebPlotDigitizer (version 4.x) (35). Each graph was digitized twice by separate reviewers to minimize measurement error, and the averaged values were used in subsequent analyses.

*Pre–post correlation assumption:* As most studies did not report the correlation between baseline and post-fasting measures, a conservative correlation coefficient of r = 0.5 was assumed for all calculations involving within-subject standardized mean differences. To assess the robustness of this assumption, sensitivity analyses were additionally performed using alternative plausible correlation values (r = 0.3 and r = 0.7).

*Fasting duration standardization:* In this meta-analysis the post- timepoint corresponds to the end-of-fast. In all included studies, the “next” time point consistently refers to measurements obtained at the end of the fasting period, while participants were still fasting and before refeeding. When multiple time points were reported in a study, the last measurement obtained at the end of the fasting period was selected for analysis. Post-refeeding measurements were excluded to ensure comparability between studies and to isolate the metabolic effects of fasting.

Fasting duration was harmonized across studies by converting all reported units (hours, days, or ranges) into a single continuous variable expressed in days. For range values (e.g., “3–5 days”), the midpoint was used. To address potential non-independence arising from multiple fasting durations or study arms within individual trials, an additional dataset was generated in which only the longest fasting duration per study and biomarker was retained (“longest-only” dataset). This dataset was used in parallel sensitivity analyses to evaluate the stability of pooled estimates and threshold parameters.

All transformed and standardized data were compiled into a single harmonized dataset and underwent internal quality checks before meta-analysis.

### 2.4 Effect size calculation

The effect size for each study was expressed as Hedges’ g, representing the bias-corrected standardized mean difference between post-fasting and pre-fasting lipid concentrations. This metric accounts for within-subject dependency and corrects for small-sample bias, making it suitable for pre–post intervention designs.

For each study, Hedges’ g was calculated as:


$$g=J\times \frac{M_{\text{post}}-M_{\mathrm{pre}}\;\;}{SD_{\text{change}}}$$

Where *M_post_* and *M_pre_* denote the mean lipid values after and before fasting, respectively, and $J=1-\frac{3}{4\mathrm{df}-1}$ is the small-sample correction factor.

The standard deviation of the pre–post change scores was computed as:


$$SD_{\text{change}}=\sqrt{S{D_{\mathrm{pre}}}^{2}+S{D_{\text{post}}}^{2}-2r\cdot (SD_{\mathrm{pre}})\cdot (SD_{\text{post}})}$$

Where r represents the within-subject pre–post correlation. Because most studies did not report this correlation, the primary analyses assumed r = 0.5. To assess robustness to this assumption, sensitivity analyses were additionally conducted using r = 0.3 and r = 0.7.

When a study reported multiple fasting subgroups, durations, or measurement time points, within-study pooling was performed to derive a single representative effect size per biomarker. This was achieved through inverse-variance weighting:


$$g_{\text{combined}}=\frac{\sum w_{i}g_{i}\;}{\sum w_{i}},\text{where}\;w_{i}=\frac{1\;}{v_{i}}$$

Because this approach may still introduce residual dependence, additional sensitivity analyses were conducted using a “longest-only” dataset retaining a single time point per study.

All computations were conducted using custom Python scripts developed for this meta-analysis. Effect sizes and their corresponding variances were subsequently entered into the random-effects models described in the next section.

### 2.5 Statistical analysis

All statistical analyses were conducted following the methodological recommendations for quantitative synthesis of pre–post intervention studies. Each lipid biomarker — including total cholesterol (TC), low-density lipoprotein (LDL), high-density lipoprotein (HDL), very-low-density lipoprotein (VLDL), and triglycerides (TG) — was analyzed separately.

*Overall meta-analysis:* The pooled effect size for each biomarker was calculated using a random-effects model based on the DerSimonian–Laird (DL) method. This approach accounts for both within-study and between-study variability, thereby providing a more conservative and generalizable estimate of the fasting effect on lipid levels. To evaluate the robustness of between-study variance estimation, additional random-effects models were fitted using restricted maximum likelihood (REML) and Paule–Mandel (PM) estimators. Consistency of pooled effects across τ^2^ estimators was examined as part of sensitivity analyses.

The pooled estimate ( $g_{\mathrm{RE}}$ ) and its variance were computed as:


$$g_{\mathrm{RE}}=\frac{\sum w{*}_{i}g_{i}\;}{\sum w{*}_{i}},\text{where}\;w{*}_{i}=\frac{1\;}{v_{i}+\tau^{2}}$$

Here, $v_{i}$ is the within-study variance, and *τ^2^* represents the between-study variance estimated using the DL method.

Between-study heterogeneity was quantified using Cochran’s Q statistic, *I^2^*, and *τ^2^* values:


$$I^{2}=\frac{Q-(k-1)\;}{Q}\times 100\%$$

Where *k* is the number of studies. Values of I^2^ around 25, 50, and 75% were interpreted as low, moderate, and high heterogeneity, respectively.

Sensitivity analyses were performed to confirm the stability of pooled estimates across alternative τ^2^ estimators and correlation assumptions.

*Subgroup analysis:* To examine whether fasting duration moderated lipid responses, studies were stratified into two predefined duration categories (≤3 days vs. > 3 days) based on the empirically derived breakpoint suggested by the threshold meta-regression models. Each subgroup was analyzed separately using random-effects models, and between-group heterogeneity was evaluated using the Q < sub > Between</sub > statistic (*χ*^2^ distribution). A statistically significant Q < sub > Between</sub > (*p <* 0.05) was interpreted as evidence of duration-dependent moderation. This binary categorization was implemented to align categorical subgroup comparisons with the continuous threshold meta-regression framework and to avoid sparse data issues associated with multi-level stratification. Subgroup-specific and pooled forest plots were generated for each biomarker.

*Threshold meta-regression analysis:* Threshold Meta-Regression Analysis: In addition to categorical analysis, a threshold meta-regression was conducted to identify potential non-linear relationships between fasting duration (continuous moderator, expressed in days) and lipid responses. A piecewise (segmented) meta-regression model was fitted using inverse-variance weighting under a random-effects framework. The model was defined as:


$$g_{i}=\beta_{0}+\beta_{1}\cdot \mathrm{Day}s_{i}+\beta_{2}\cdot \max\;(0,\mathrm{Day}s_{i}-\tau )+\in_{i}$$

Where *β*₁ represents the slope before the threshold (*τ*), and (*β*₁ + β₂) represents the slope after the threshold.

The breakpoint (τ) was estimated through an iterative grid-search procedure across the observed range of fasting durations, selecting the value that minimized residual heterogeneity (Q_E). A statistically significant coefficient for β₂ (*p <* 0.05) indicated a change in slope beyond the threshold, consistent with a duration-dependent effect. To quantify uncertainty around the estimated threshold, non-parametric bootstrap resampling (1,000 iterations) was performed, and 95% confidence intervals for *τ* were derived. Influence diagnostics were additionally conducted using leave-one-out analyses to examine the stability of the breakpoint and slope estimates across individual studies. All threshold analyses were conducted separately for each lipid biomarker.

*Visualization and reporting:* Forest plots were used to display individual study effect sizes and pooled estimates with corresponding 95% confidence intervals for each lipid biomarker. Funnel plots were generated to visually assess potential asymmetry. All figures were produced in a black-and-white format to ensure visual consistency across biomarkers.

*Statistical software:* All analyses were performed using Python (version 3.10) with custom-written scripts utilizing the pandas, numpy, scipy.stats, and matplotlib libraries. Random-effects modeling, subgroup comparisons, threshold meta-regression, bootstrap confidence interval estimation, and leave-one-out influence analyses were implemented within this unified analytical pipeline in accordance with established meta-analytic principles.

### 2.6 Publication bias assessment

Potential publication bias was evaluated using both visual and statistical approaches. Funnel plots were generated for each lipid biomarker to examine the symmetry of effect size distributions relative to study precision (standard error). In the absence of bias, studies are expected to form a symmetrical inverted funnel around the pooled effect size. Funnel plot asymmetry was statistically assessed using Egger’s regression intercept test, which examines the association between standardized effect sizes and their standard errors. A significant intercept (*p <* 0.05) was considered indicative of possible small-study effects or selective publication bias. Given the limited number of studies for certain biomarkers, Egger’s test results were interpreted cautiously.

### 2.7 Risk of bias considerations

Risk of bias was assessed at the study level using validated, design-specific tools. Randomized controlled trials (RCTs) were evaluated using the Cochrane Risk of Bias 2 (RoB 2) tool, while non-randomized intervention studies (NRSIs) were assessed using the ROBINS-I tool. Two reviewers independently performed all assessments, and di-sagreements were resolved by consensus.

Certainty of evidence was subsequently evaluated at the outcome level using the GRADE framework. The following domains were considered: risk of bias, inconsistency, indirectness, imprecision, and publication bias. Given that the majority of included studies were non-randomized single-arm interventions, evidence was initially rated as low certainty and downgraded where appropriate. Detailed risk-of-bias assessments and GRADE Summary of Findings tables are provided in the Supplementary Figures S1, S2 and Supplementary Tables S2).

### 2.8 Software and reproducibility

All statistical computations, data processing, and visualizations were performed using Python (version 3.10) within a fully reproducible analytical environment. The workflow integrated the following packages: pandas (data manipulation), numpy (numerical computation), scipy.stats (statistical analysis), and matplotlib (data visualization). Custom-built Python scripts were used to perform data standardization, random-effects meta-analysis, subgroup comparisons, and threshold meta-regression. To enhance transparency and reproducibility, all analytic procedures, parameter definitions, and transformation rules were documented step by step. The analysis code and example datasets will be made available upon reasonable request to the corresponding author, allowing full replication of the meta-analytic process.

## 3 Results

### 3.1 Study selection

**Figure 1:**
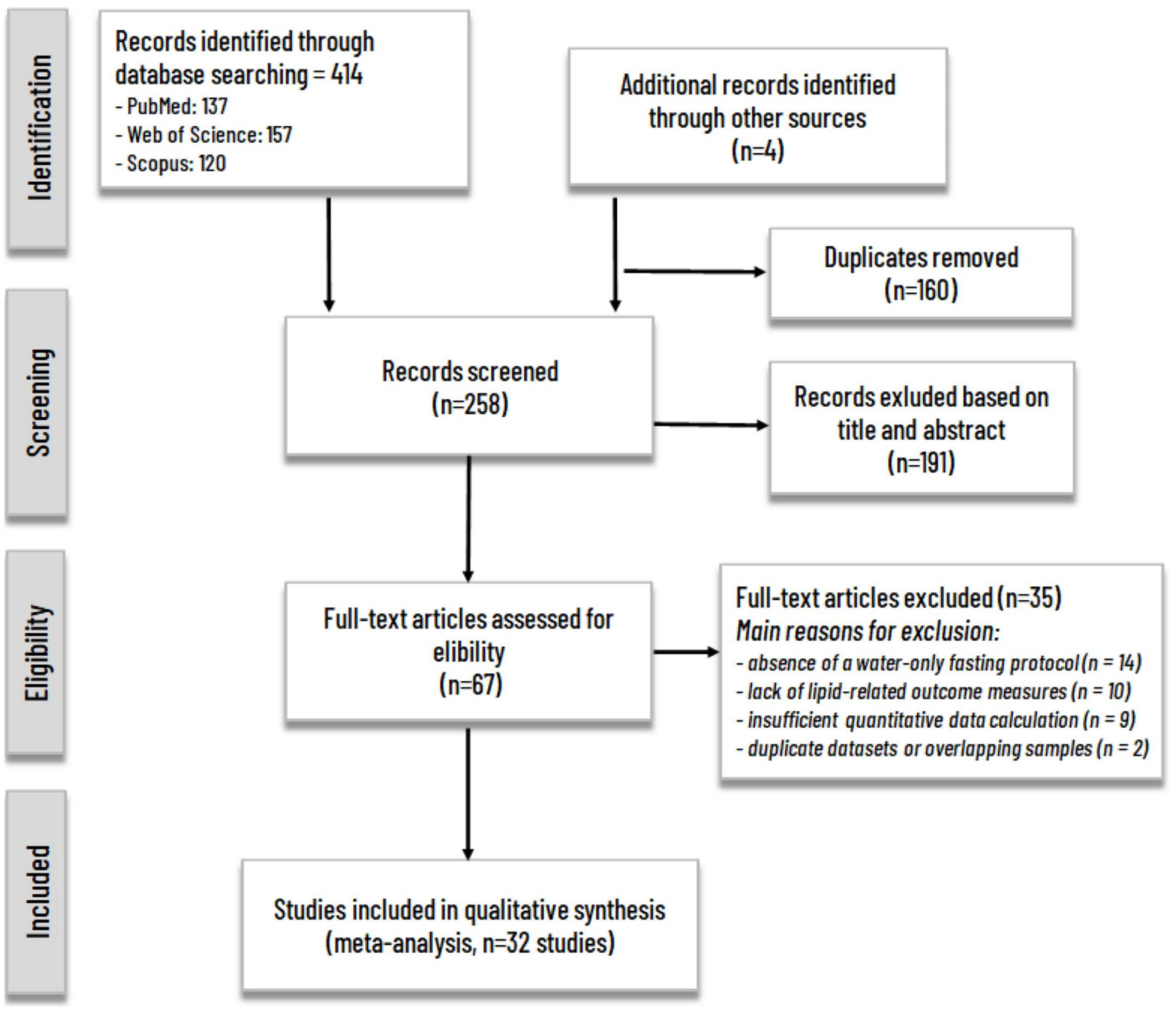
PRISMA flowchart of study search and selection process.

The comprehensive literature search identified 418 records in total: 414 from electronic databases (PubMed = 137, Scopus = 120, Web of Science = 157) and 4 additional studies from manual searches of reference lists and other relevant sources. After removing duplicates, 258 unique studies were screened based on titles and abstracts. Of these, 67 full-text articles were assessed for eligibility, and 32 studies met the inclusion criteria and were included in the quantitative synthesis (meta-analysis). The study selection process is summarized in the PRISMA 2020 flow diagram (Figure 1). Reasons for exclusion at the full-text stage included:

- absence of a water-only fasting protocol (*n =* 14),
- lack of lipid-related outcome measures (*n =* 10),
- insufficient quantitative data for effect size calculation (*n =* 9), and
- duplicate datasets or overlapping samples (*n =* 2).

The final dataset comprised 32 independent studies published between 2000 and 2025, with a cumulative sample size of approximately >600 participants. The majority of the studies employed a pre–post intervention design, while a few incorporated multiple fasting arms or randomized control elements. The included studies collectively investigated the effects of water-only fasting on serum TC, LDL, HDL, VLDL, and TG.

### 3.2 Study characteristics

After within-study pooling, a total of 109 biomarker-specific effect sizes derived from multiple fasting durations and study arms were included in the quantitative synthesis. These estimates originated from 32 studies for triglycerides, 25 for total cholesterol, 23 for HDL, 22 for LDL, and 7 for VLDL. Across all included comparisons, fasting duration ranged from 1 to 17 days, with a median duration of 4 days. Most studies enrolled generally healthy adults, although several investigations included participants with overweight, obesity, hyperlipidemia, or type 2 diabetes. Study-level characteristics and sample details are summarized in Table 1.

**Table 1:** Descriptive summary of water-only fasting included studies.

Author (year), country	Study design and population	Fasting protocol and duration	Reported lipid changes	Risk of bias/quality metrics
Arner et al. (2003) Sweden (13), [Data reported in Gälman et al. (2008) (38)]	Prospective single-arm experimental study. Sample size: 7 (3 males, 4 females). Healthy outpatient volunteers, mean age 31 ± 2 years.	48-h water-only fasting.	HDL concentrations increased from 1.40 ± 0.11 mmol/L to 1.57 ± 0.13 mmol/L. Triglyceride levels showed minimal change, from 0.69 ± 0.10 mmol/L to 0.71 ± 0.09 mmol/L. Total cholesterol exhibited a modest increase, rising from 4.16 ± 0.25 mmol/L to 4.51 ± 0.32 mmol/L following 48 h of fasting.	Moderate risk (small sample size; single-arm design; adequate protocol description).
Blondheim et al. (2001), Israel (24)	Non-randomized crossover study. Sample size: 13 volunteers (5 males, 8 females). Population: 13 normal adults (mean age 45.1 ± 12.8 years; BMI 25.1 ± 3.8 kg/m^2^).	24-h food-and-water fasting. Each participant completed three experimental conditions in a crossover manner: High-protein pre-fast meal, High-carbohydrate pre-fast meal, High-fat pre-fast meal	Total cholesterol responses to 24 h of food-and-water fasting varied according to pre-fast macronutrient composition. Following a high-protein pre-fast meal, total cholesterol showed minimal change, increasing slightly from 170.5 ± 36.7 mg/dL to 171.8 ± 34.9 mg/dL. After a high-carbohydrate pre-fast meal, concentrations demonstrated a modest increase, rising from 173.7 ± 33.7 mg/dL to 179.6 ± 36.3 mg/dL. In contrast, the high-fat pre-fast meal produced the most pronounced elevation, with levels rising from 174.0 ± 34.5 mg/dL to 187.9 ± 36.6 mg/dL. Triglyceride concentrations decreased consistently across all three pre-fast dietary conditions. After a high-protein meal, triglycerides declined from 120.7 ± 52.1 mg/dL to 85.1 ± 33.0 mg/dL. Following a high-carbohydrate meal, levels decreased from 128.5 ± 57.0 mg/dL to 91.5 ± 46.5 mg/dL. The largest reduction occurred after a high-fat meal, with triglycerides dropping from 155.1 ± 59.6 mg/dL to 95.0 ± 36.5 mg/dL.	Moderate risk (non-randomized design; small sample; crossover nature increases internal consistency).
Browning et al. (2012), United States (14)	Prospective single-arm interventional metabolic study. Total sample: 18 healthy adults (9 females, 9 males). Women were pre-menopausal. Median ages: women 24 years (23–42), men 21 years (21–23).	48-h extended fasting (48-h fast).	“Among women (*n =* 9), total cholesterol increased from 167 mg/dL (163–184) to 176 mg/dL (154–195). Among men (*n =* 9), total cholesterol increased from 164 mg/dL (155–194) to 166 mg/dL (161–193).” “Among women (*n =* 9), triglyceride levels increased from 67 mg/dL (58–79) to 75 mg/dL (75–110). Among men (*n =* 9), triglyceride levels increased from 52 mg/dL (50–94) to 75 mg/dL (65–85).”	Moderate risk (single-arm design; small sex-specific subgroups; metabolic study setting).
Canavan et al. (2005), USA (22)	Randomized, double-blinded, placebo-controlled study. Sample size: 20 females. Population: Twenty healthy, normal-weight women of reproductive age.	4-day water-only fasting.	Triglycerides increased markedly, rising from 74 ± 7 mg/dL to 114 ± 9 mg/dL.	Low to moderate risk (randomized, double-blind, placebo-controlled design provides strong methodological rigor; sample limited to females).
Commissati et al. (2025), Australia/United States (study center) (15)	Prospective single-arm interventional experimental human study. Total sample: 20 volunteers (9 males, 11 females). Middle-aged adults with mean age 52.2 ± 11.8 years and BMI 28.8 ± 6.4 kg/m^2^. HDL analysis included *n =* 19 participants.	Prolonged fasting for ~10 days (mean duration 9.8 ± 3.1 days).	HDL concentrations decreased from 56.8 ± 19.2 mg/dL to 47.8 ± 12.3 mg/dL. LDL-C increased from 108.4 ± 37.4 mg/dL to 131.6 ± 55.5 mg/dL. Triglyceride levels increased from 102.8 ± 46.2 mg/dL to 125.4 ± 37.4 mg/dL. Total cholesterol increased from 192.0 ± 33.5 mg/dL to 216.6 ± 47.8 mg/dL.	Moderate risk (single-arm design; adequate reporting; moderate sample size).
Dai et al. (2022), China (5)	Prospective single-arm experimental human study. Sample size: 13 males. Mean age 39.6 ± 7.9 years (range 28–55), mean weight 72.1 ± 11.9 kg, BMI 24.6 ± 3.5 kg/m^2^ (range 19.2–31.9).	10-day prolonged fasting (water-only).	HDL concentrations decreased from 1.14 ± 0.27 mmol/L to 0.97 ± 0.22 mmol/L. LDL-C increased from 3.67 ± 0.85 mmol/L to 5.05 ± 1.05 mmol/L. Triglyceride levels decreased from 1.90 ± 1.08 mmol/L to 1.49 ± 0.30 mmol/L. Total cholesterol increased from 4.88 ± 0.76 mmol/L to 6.46 ± 1.16 mmol/L.	Moderate risk (single-arm design; adequate participant characterization; no control group).
Fainaru and Schafer (2000), Israel (31)	Observational study. Total sample: 12 physicians (8 males, 4 females), aged 25–59 years. Participants were divided into four subgroups: (1) normolipidemic non-obese men (*n =* 4), (2) moderately obese or type IV hyperlipidemic men (*n =* 4), (3) healthy non-obese women (*n =* 2), (4) women on oral contraceptives (*n =* 2).	Prolonged fasting for 3–5 days during a monitored hunger strike.	On fasting Day 3 (*n =* 12), HDL concentrations decreased from 41.83 ± 9.04 mg/dL to 40.83 ± 7.41 mg/dL. On fasting Day 5 (*n =* 6), HDL decreased from 41.17 ± 7.44 mg/dL to 36.00 ± 6.84 mg/dL. On fasting Day 3 (*n =* 12), LDL concentrations increased from 147.33 ± 37.42 mg/dL to 154.58 ± 46.42 mg/dL. On fasting Day 5 (*n =* 5), LDL increased from 138.60 ± 47.99 mg/dL to 160.20 ± 68.51 mg/dL. On fasting Day 3 (*n =* 12), triglyceride levels changed minimally, from 95.75 ± 76.33 mg/dL to 96.67 ± 42.98 mg/dL. On fasting Day 5 (*n =* 5), triglycerides decreased from 109.20 ± 88.70 mg/dL to 103.00 ± 45.29 mg/dL. On fasting Day 3, VLDL decreased from 17.00 ± 19.13 mg/dL to 15.08 ± 10.33 mg/dL. On fasting Day 5, VLDL decreased from 23.20 ± 24.81 mg/dL to 17.20 ± 10.85 mg/dL. On fasting Day 3, total cholesterol increased from 206.50 ± 46.40 mg/dL to 211.42 ± 52.23 mg/dL, and on fasting Day 5, it increased from 203.40 ± 65.92 mg/dL to 214.00 ± 73.76 mg/dL.	Moderate risk (observational design; heterogeneous subgroups; non-controlled conditions).
Fang et al. (2021), China (16)	Longitudinal study. Total sample: 31 participants (11 males, 20 females), aged 27–67 years. Meta-analysis included HDL subgroups of normal-HDL males (*n =* 7), normal-HDL females (*n =* 10), low-HDL males (*n =* 4), and low-HDL females (*n =* 1).	7-day water-only fasting.	1. HDL HDL concentrations decreased in most subgroups. Normal-HDL males: 1.183 mmol/L to 0.931 mmol/L Normal-HDL females: 1.278 mmol/L to 1.240 mmol/L Low-HDL males: 0.938 mmol/L to 0.793 mmol/L Low-HDL females: 1.010 mmol/L to 1.450 mmol/L 2. LDL LDL concentrations increased across all subgroups. Normal-HDL males: 2.75 mmol/L to 4.51 mmol/L Normal-HDL females: 2.32 mmol/L to 3.77 mmol/L High-LDL males: 4.24 mmol/L to 6.95 mmol/L High-LDL females: 3.75 mmol/L to 5.70 mmol/L 3. Triglycerides Triglyceride responses varied by subgroup. Normal-TG males: 1.172 mmol/L to 1.417 mmol/L Normal-TG females: 0.959 mmol/L to 1.352 mmol/L High-TG males: 2.788 mmol/L to 1.713 mmol/L High-TG females: 2.027 mmol/L to 1.283 mmol/L 4. Total Cholesterol Total cholesterol increased in all subgroups. Normal-TC males: 4.408 mmol/L to 6.046 mmol/L Normal-TC females: 4.201 mmol/L to 5.608 mmol/L High-TC males: 6.476 mmol/L to 8.580 mmol/L High-TC females: 5.697 mmol/L to 7.647 mmol/L	Moderate risk (single-arm longitudinal design; adequate reporting).
Gabriel et al. (2022), United States (4)	Single-arm, open-label observational study. Sample size: 38 (7 male, 31 female). Non-diabetic, overweight/obese adults aged 40–70 years. Median age: 60 (IQR 52–65). BMI: 25–40 kg/m^2^.	Water-only fasting for ≥10 consecutive days (median duration 14 days, IQR 13–19).	HDL concentrations decreased, with the median changing from 1.29 (1.04–1.62) mmol/L to 1.15 (0.96–1.30) mmol/L LDL concentrations increased, shifting from 3.17 (2.51–3.76) mmol/L to 3.28 (2.65–3.89) mmol/L VLDL concentrations increased from 0.49 mmol/L (IQR: 0.44–0.64) to 0.60 mmol/L (IQR: 0.54–0.69) following at least 10 days of water-only fasting (*n =* 38) Total cholesterol remained stable, changing from 5.10 (4.38–5.56) mmol/L to 5.06 (4.36–5.55) mmol/L Triglyceride levels increased, with the median changing from 1.16 (1.05–1.56) mmol/L to 1.48 (1.29–1.65) mmol/L	Low–moderate risk (observational design; single-arm; adequate reporting).
Gonzalez and Cooke (2022), Michigan, USA (8)	Randomized controlled crossover design. Sample size: 25 participants (14 male, 11 female). Population: Twenty-five healthy young adults (age 23 ± 3 yr.; height 176 ± 16 cm; weight 76 ± 16 kg; BMI 24 ± 4 kg/m^2^; BSA 1.9 ± 0.2 m^2^).	24-h complete fasting.	HDL cholesterol showed a slight decrease, falling from 58.8 ± 18.9 mg/dL to 56.2 ± 20.3 mg/dL. LDL cholesterol increased notably after 24 h of fasting, rising from 95.6 ± 29.9 mg/dL to 105.5 ± 21.5 mg/dL. Total cholesterol remained essentially unchanged, shifting only from 172.4 ± 35.2 mg/dL to 172.7 ± 29.1 mg/dL. Triglyceride levels decreased substantially, falling from 120.8 ± 61.6 mg/dL to 77.2 ± 37.9 mg/dL.	Low to moderate risk (well-controlled crossover design; healthy homogeneous population; adequate methodological description).
Horne et al. (2013), USA (11)	Randomized cross-over trial. Total sample: 30 (20 female, 10 male). Sub-analysis fasting-first group: *n =* 16. Apparently healthy adults, mean age 43.6 ± 13.5 years (range 19.5–64.4).	Water-only fasting for 24 h, compared with a 24-h ad libitum control period.	HDL concentrations increased from 54.4 ± 12.1 mg/dL to 57.4 ± 12.1 mg/dL following 24 h of water-only fasting. LDL-C increased by 23.1 ± 35.1 mg/dL after 24 h of water-only fasting. Total cholesterol increased from 192 ± 27 mg/dL to 200 ± 32 mg/dL after 24 h of water-only fasting. Triglyceride levels decreased from 132 ± 46 mg/dL to 94 ± 33 mg/dL.	Low risk of bias (randomized cross-over design; good methodological control).
Jiang et al. (2021), China (17)	Prospective single-arm clinical trial. Sample size: 41 (17 male, 24 female). Participants were healthy and within the normal weight range.	5-day water-only fast.	HDL concentrations decreased from 1.56 ± 0.37 mmol/L to 1.38 ± 0.33 mmol/L. LDL-C increased from 3.65 ± 0.96 mmol/L to 5.33 ± 1.16 mmol/L. Triglyceride levels increased from 1.17 ± 0.88 mmol/L to 1.68 ± 0.62 mmol/L. Total cholesterol increased from 5.56 ± 1.03 mmol/L to 7.46 ± 1.36 mmol/L.	Moderate risk (single-arm design; acceptable reporting quality).
Jørgensen et al. (2015), Denmark (25)	Prospective clinical study. Total sample: 39 young healthy white men (21 SGA, 18 AGA). HDL sub-analysis included *n =* 18 participants.	36-h water-only fasting (water allowed ad libitum). Smokers were permitted to use a 10 mg Nicorette inhaler.	HDL concentrations increased from 1.13 ± 0.27 mmol/L to 1.15 ± 0.27 mmol/L; the study also reported SE-based values of 1.13 ± 0.07 mmol/L to 1.15 ± 0.07 mmol/L. LDL-C increased from 2.69 ± 0.15 mmol/L to 2.78 ± 0.15 mmol/L. Triglyceride levels decreased from 1.04 ± 0.08 mmol/L to 0.93 ± 0.07 mmol/L. Total cholesterol increased from 4.31 ± 0.17 mmol/L to 4.37 ± 0.17 mmol/L following 36 h of fasting.	Moderate risk of bias (single-arm clinical design; adequate methodological reporting).
Letkiewicz et al. (2020), Poland (49)	Prospective single-arm pre–post interventional study. Sample size: 14 healthy men, aged 35–60 years (mean 49.64 ± 9.30, median 52).	8-day water-only fasting.	Total cholesterol increased from 212.21 ± 60.00 mg/dL to 225.28 ± 76.21 mg/dL.	Moderate risk (single-arm design; small homogeneous sample; clear protocol description).
Mojto et al. (2018), Slovakia (28)	Prospective single-arm interventional clinical study. Sample size: 10 participants (5 males, 5 females). Men: mean age 46.4 years (31–58), women: mean age 53.6 years (44–63).	11-day water-only fasting.	HDL concentrations decreased from 1.468 ± 0.09 mmol/L to 1.164 ± 0.08 mmol/L. LDL-C increased from 4.44 ± 0.41 mmol/L to 4.93 ± 0.48 mmol/L. Triglyceride levels remained stable, changing from 1.086 ± 0.17 mmol/L to 1.095 ± 0.09 mmol/L. Total cholesterol increased from 5.892 ± 0.39 mmol/L to 6.159 ± 0.46 mmol/L.	Moderate risk (small sample, single-arm design, adequate reporting).
Moser et al. (2021), Austria (12)	Single-center, cross-over controlled clinical trial. Sample size: 20 adults with type 1 diabetes (13 males, 7 females). Mean age: 35 ± 11 years.	36-h prolonged fasting (baseline measurement after 12 h fasting; post measurement after 36 h fasting).	HDL concentrations decreased from 73 ± 18 mg/dL to 71 ± 20 mg/dL. LDL concentrations increased from 102 ± 34 mg/dL to 109 ± 32 mg/dL. Triglyceride levels increased from 64 ± 18 mg/dL to 80 ± 28 mg/dL. VLDL increased from 14 ± 4 mg/dL to 17 ± 4 mg/dL. Total cholesterol increased from 195 ± 32 mg/dL to 201 ± 34 mg/dL.	Low–moderate risk (controlled cross-over design; clinical population; robust methodology).
Nuttall et al. (2020), USA (30)	Randomized crossover study. Sample size: 7 males. Population: Seven adult male participants with type 2 diabetes mellitus.	72-h prolonged fasting (water-only).	HDL cholesterol decreased slightly, falling from 1.0 ± 0.10 mmol/L to 0.9 ± 0.10 mmol/L. LDL-C increased slightly after 72 h of prolonged fasting, rising from 2.3 ± 0.03 mmol/L to 2.5 ± 0.44 mmol/L. Total cholesterol remained unchanged, with values stable from 4.0 ± 0.38 mmol/L to 4.0 ± 0.44 mmol/L. Triacylglycerol (TAG) concentrations decreased slightly, changing from 1.5 ± 0.1 mmol/L to 1.4 ± 0.1 mmol/L. VLDL concentrations decreased slightly, declining from 0.7 ± 0.10 mmol/L to 0.6 ± 0.03 mmol/L.	Moderate risk (very small sample size; T2DM population; randomized crossover design provides methodological strength).
Pietzner et al. (2024), Germany (39)	Prospective longitudinal interventional study. Sample size: 12 participants (7 male, 5 female). Population: 12 apparently healthy, non-smoking adults of predominantly white-European ancestry. Exclusion criteria: Any known disease, percent body fat <15% for females or <12% for males.	7-day water-only fasting.	HDL cholesterol decreased, falling from 1.40 ± 0.43 mmol/L to 1.23 ± 0.38 mmol/L. LDL concentrations increased during the 7-day water-only fast, rising from 2.85 ± 0.91 mmol/L to 3.30 ± 0.79 mmol/L. Triglyceride concentrations also increased, rising from 0.81 ± 0.37 mM to 1.15 ± 0.29 mM.	Moderate risk (small sample size; single-arm interventional design; adequate participant screening).
Pilis et al. (2023), Poland (6)	Prospective single-arm, within-subject experimental intervention study. Sample size: 16 healthy male volunteers, mean age 53.38 ± 13.73 years, white-collar workers.	8-day water-only fasting.	At rest, HDL concentrations increased from 52.63 ± 8.97 mg/dL to 53.56 ± 8.70 mg/dL. After exercise, HDL increased from 57.38 ± 9.96 mg/dL to 58.56 ± 10.01 mg/dL. At rest, LDL-C changed from 146.9 ± 51.6 mg/dL to 148.4 ± 66.4 mg/dL. After exercise, LDL-C changed from 160.1 ± 53.0 mg/dL to 156.28 ± 66.9 mg/dL. Triglyceride levels increased from 106.2 ± 64.6 mg/dL to 110.5 ± 30.6 mg/dL. Under resting conditions, total cholesterol increased from 220.8 ± 60.7 mg/dL to 223.9 ± 71.65 mg/dL. Under exercise conditions, total cholesterol changed from 241.7 ± 61.3 mg/dL to 241.25 ± 74.8 mg/dL.	Moderate risk (single-arm design; small sample; good within-subject control).
Pilis et al. (2025), Poland (50)	Prospective single-group experimental intervention study. Sample size: 13 healthy middle-aged men (mean age 57.25 ± 9.93 years, mean weight 81.72 ± 9.19 kg, height 178.54 ± 4.77 cm).	8-day water-only fasting combined with exercise.	Under resting conditions, triglyceride levels increased from 90.23 ± 33.75 mg/dL to 112.54 ± 28.10 mg/dL. Under exercise conditions, triglyceride levels increased from 105.46 ± 36.68 mg/dL to 136.23 ± 27.17 mg/dL.	Moderate risk (single-group design; small homogeneous sample; combined fasting–exercise protocol).
Quiroz-Olguín et al. (2025), Mexico (20)	Proof-of-concept study. Sample size: 11 participants (7 males, 4 females). Volunteers with a median age of 43 years (IQR: 40–56).	72-h complete fast (no food and no water for 3 days).	HDL concentrations decreased, with the median changing from 24.1 mg/dL (17.3–38.2) to 20.6 mg/dL (16.6–29.9). LDL-C increased from 109.2 mg/dL (95.1–126.8) to 131.0 mg/dL (111.0–160.8). Triglyceride levels decreased from 191.1 mg/dL (94.9–237.5) to 116.9 mg/dL (81–125). Total cholesterol increased from 170.6 mg/dL (147.8–208.1) to 179.3 mg/dL (155.1–222.6).	High risk (small sample size; extreme fasting protocol without water; proof-of-concept design).
Reingold et al. (2005), Texas, USA (51)	Prospective within-subject experimental study. Sample size: 7 healthy volunteers (3 males, 4 females). Mean BMI: 25 ± 4 kg/m^2^.	48-h water-only fasting.	Triglyceride levels decreased from 65 ± 11 mmol/L to 55 ± 10 mmol/L.	Moderate risk (very small sample size; within-subject design; adequate methodological control).
Rhodes et al. (2023), USA (California) (21)	Rigorously controlled pilot clinical study. Sample size: 20 young healthy adults (10 males, 10 females). Mean age 27.5 ± 4.35 years.	36-h prolonged fasting.	HDL concentrations increased from 66.1 ± 16.8 mg/dL to 67.7 ± 15.1 mg/dL. LDL concentrations increased from 77.5 ± 27.0 mg/dL to 85.8 ± 25.1 mg/dL. Triglyceride levels decreased from 94.4 ± 31.6 mg/dL to 81.8 ± 17.8 mg/dL. Total cholesterol increased from 163.1 ± 36.3 mg/dL to 166.8 ± 29.2 mg/dL.	Low–moderate risk (well-controlled environment, small sample size typical for pilot studies).
Rubio-Aliaga et al. (2011), Scotland (32)	Single-arm, repeated-measures experimental study. Sample size: 10 participants (3 males, 7 females). Heterogeneous volunteer group with wide ranges of BMI (18.5–39.7 kg/m^2^), age (25–56 years), and fasting glucose (86–119 mg/dL).	36-h prolonged fasting.	HDL concentrations changed minimally, from 48.52 ± 7.93 mg/dL to 48.60 ± 9.47 mg/dL. LDL-C increased from 93.35 ± 18.57 mg/dL to 98.80 ± 17.72 mg/dL. Triglyceride levels decreased from 132.97 ± 96.07 mg/dL to 88.60 ± 21.28 mg/dL. VLDL decreased from 5.72 ± 6.50 mg/dL to 2.70 ± 1.83 mg/dL. Total cholesterol increased from 147.38 ± 24.15 mg/dL to 150.10 ± 21.79 mg/dL.	Moderate risk (small heterogeneous sample; single-arm repeated-measures design).
Ruge et al. (2001), Sweden (26)	Single-group, within-subject experimental study. Sample size: 14 healthy volunteers (4 males, 10 females). Participants were university students or employees, regularly performing fitness activity (ball games, long walks, or aerobic training) 3–4 times per week. Age range 19–48 years.	30-h water-only fasting.	Triglyceride levels decreased from 2.5 ± 0.6 mmol/L to 0.86 ± 0.1 mmol/L.	Moderate risk (single-group design; small sample; physically active volunteers).
Ruge et al. (2005), Sweden (27)	Single-group, within-subject experimental study. Total study sample: 28 healthy young adults (17 females, 9 males). Mean age 28 ± 2 years, BMI 23 ± 1 kg/m^2^. Triglyceride analysis included *n =* 12 participants, although baseline data were reported for 28 individuals.	30-h water-only fasting.	Triglyceride levels decreased from 1.5 ± 0.2 mmol/L to 1.0 ± 0.1 mmol/L.	Moderate risk (sample inconsistency between reported values and analyzed subgroup; single-group design).
Sanchetee et al. (2020), India (7)	Prospective study. Total sample: 110 participants (27 males, 83 females), age range 13–86 years (mean age 50.2 years).	Water-only fasting for 3–30 days. Subgroups: short fast 3–7 days (*n =* 72, mean 3.4 days); prolonged fast >7 days (*n =* 38, mean 10.1 days).	HDL concentrations decreased from 48.3 ± 12.2 mg/dL to 45.7 ± 12.6 mg/dL. LDL-C remained unchanged at 132.8 ± 37.8 mg/dL before and after fasting. Triglyceride levels increased from 137.6 ± 68.3 mg/dL to 149.0 ± 67.1 mg/dL. VLDL increased from 22.0 ± 10.5 mg/dL to 24.2 ± 11.1 mg/dL. Total cholesterol changed minimally, from 203.0 ± 46.9 mg/dL to 203.9 ± 49.1 mg/dL.	Moderate risk (large sample size but heterogeneous ages; non-randomized design).
Sang et al. (2023), China (18)	Prospective longitudinal single-arm interventional study. Sample size: 46 obese adult volunteers.	7-day water-only fasting.	HDL cholesterol decreased slightly, with median values declining from 1.35 mmol/L (IQR: 1.05–1.64) to 1.24 mmol/L (IQR: 0.97–1.51). LDL cholesterol increased substantially during the 7-day water-only fast, rising from 3.32 mmol/L (IQR: 2.39–4.27) to 4.88 mmol/L (IQR: 3.46–6.27). Total cholesterol increased, with median values rising from 5.58 mmol/L (IQR: 4.55–6.65) to 6.68 mmol/L (IQR: 5.27–8.09). Triglycerides decreased during the 7-day water-only fast, falling from 2.04 mmol/L (IQR: 0.59–3.49) to 1.27 mmol/L (IQR: 0.94–1.61).	Moderate risk (single-arm design; obese population; adequate fasting protocol and participant description).
Scharf et al. (2022), USA (3)	Prospective, single-center study. Sample size: 26 participants (6 males, 20 females). Overweight/obese, non-diabetic adults.	Water-only fasting for ≥10 consecutive days (median duration 17 days).	HDL concentrations decreased from 49.3 ± 12.6 mg/dL to 43.6 ± 9.3 mg/dL. LDL concentrations increased from 135.0 (34.8) mg/dL to 139.0 (49.6) mg/dL. Triglyceride levels changed minimally, from 128.2 ± 51.5 mg/dL to 127.3 ± 22.6 mg/dL. VLDL changed minimally, from 25.7 ± 10.3 mg/dL to 25.5 ± 4.5 mg/dL following at least 10 days of water-only fasting. Total cholesterol changed from 210.0 ± 39.1 mg/dL to 208.2 ± 50.1 mg/dL.	Moderate risk (single-arm design; adequate fasting protocol; well-reported outcomes).
Snel et al. (2012), The Netherlands (19)	Randomized, controlled, crossover intervention study. Sample size: 12 healthy Caucasian males, aged 18–30 years (mean age: 22). BMI range: 20–25 kg/m^2^ (mean 22.5). All had fasting glucose < 6.1 mmol/L.	60-h prolonged fasting (water-only).	HDL concentrations decreased from 1.42 ± 0.08 mmol/L to 1.25 ± 0.07 mmol/L. LDL-C increased from 2.38 ± 0.15 mmol/L to 2.58 ± 0.19 mmol/L. Total cholesterol increased from 4.22 ± 0.18 mmol/L to 4.42 ± 0.20 mmol/L. Triglyceride levels increased from 0.94 ± 0.08 mmol/L to 1.33 ± 0.11 mmol/L.	Low risk of bias (randomized crossover design, well-controlled protocol).
Stannard et al. (2002), Australia (23)	Prospective single-group, pre–post experimental human study. Sample size: 6 healthy, physically fit nondiabetic males. Mean age: 35 years (range 23–55).	72-h water-only fasting (total fasting period reported as 84 h including preparatory phase).	Triglyceride levels increased from 0.76 ± 0.08 mmol/L to 1.16 ± 0.19 mmol/L.	Moderate risk (very small sample; single-group design; physically fit volunteers).
Yang et al. (2021), China (29)	Prospective within-subject experimental human study. Sample size: 13 males. Healthy volunteers aged 28–55 years (mean age 39.62 ± 8.13).	10-day prolonged fasting (water-only).	HDL concentrations decreased from 1.1 ± 0.3 mmol/L to 1.0 ± 0.2 mmol/L. LDL-C increased from 3.6 ± 1.0 mmol/L to 5.1 ± 1.1 mmol/L. Triglyceride levels decreased from 1.9 ± 1.1 mmol/L to 1.5 ± 0.3 mmol/L. Total cholesterol increased from 4.9 ± 0.8 mmol/L to 6.5 ± 1.2 mmol/L.	Moderate risk (single-arm design, small sample size, adequate reporting).

#### 3.2.1 Fasting protocols and duration

Across included comparisons, fasting duration ranged from 1 to 30 days, with a median duration of 4 days. Consistent with the finalized analytical framework, fasting exposures were categorized into two duration groups (≤3 days vs. > 3 days) for subgroup analyses, aligning with the empirically derived thresholds from the meta-regression models. Strict water-only fasting—defined as complete caloric abstinence with unrestricted water intake—was the dominant intervention protocol across studies. Longer fasting interventions (>3 days) were predominantly implemented within medically supervised clinical fasting programs, whereas shorter protocols (≤3 days) were typically conducted under controlled laboratory or outpatient conditions.

#### 3.2.2 Study designs and outcome measures

Most studies employed pre–post repeated-measures designs, while several adopted crossover designs in which participants completed multiple fasting conditions. After within-study pooling of multi-arm and multi-timepoint data, the final analytical dataset comprised:

- HDL: 23 independent study-level effects
- LDL: 22 independent effects
- Total cholesterol: 25 independent effects
- Triglycerides: 32 independent effects
- VLDL: 7 independent effects

This expanded dataset provided improved statistical power compared with earlier syntheses, particularly for HDL, LDL, and triglycerides. All lipid biomarkers were quantified using standard enzymatic or immunochemical assays. The combined dataset included both metabolically healthy participants and individuals with overweight, obesity, dyslipidemia, or type 2 diabetes, enabling evaluation of heterogeneous responses to fasting. Fasting exposure durations were sufficiently diverse to support both subgroup analyses and continuous threshold meta-regression. Piecewise models identified duration-dependent changes in lipid trajectories, with estimated breakpoints observed for HDL (~3 days) and LDL (~10 days), while triglyceride responses exhibited rapid early shifts without a clearly defined long-duration threshold.

### 3.3 High-density lipoprotein (HDL)

#### 3.3.1 Overall meta-analysis

**Figure 2:**
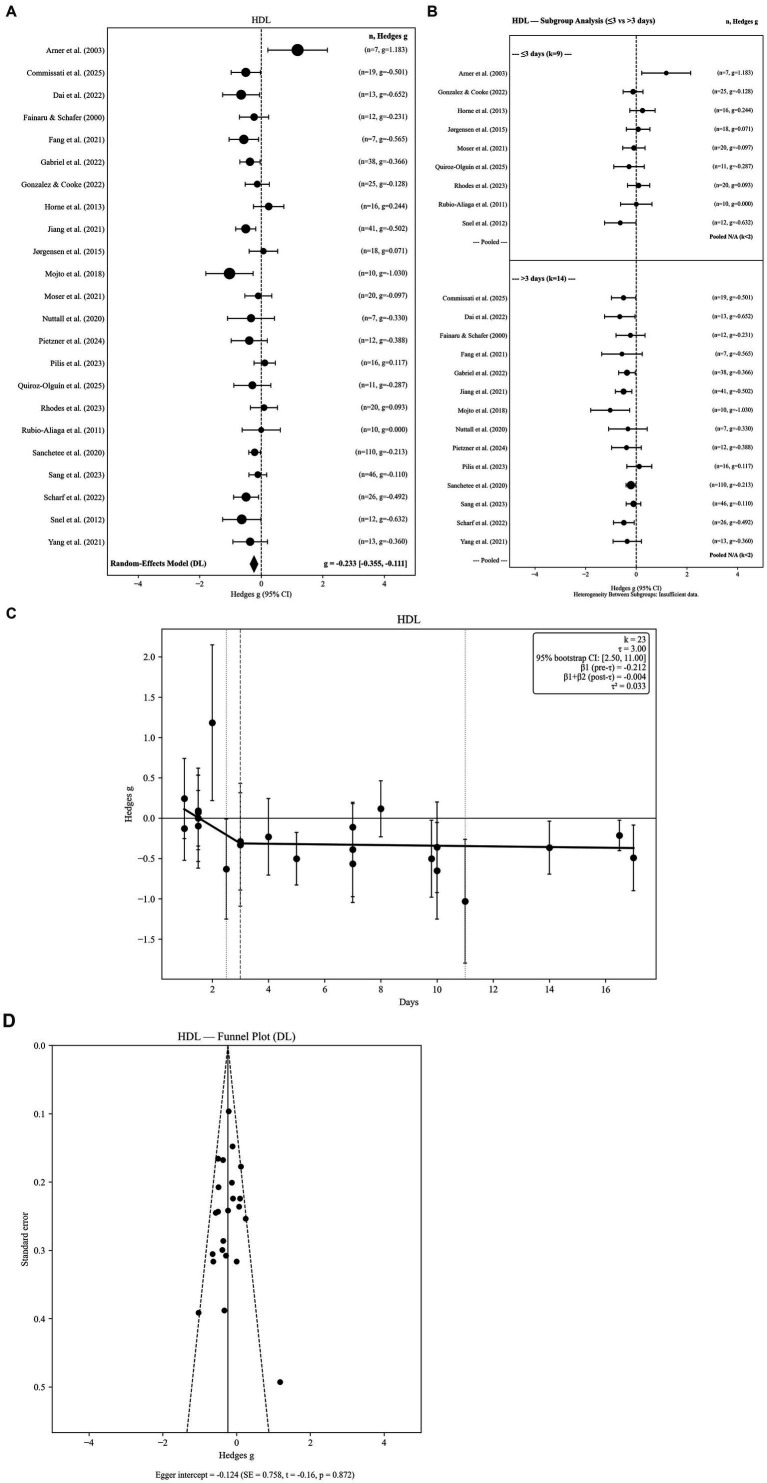
**(A)** High-density lipoprotein levels in response to fasting. **(B)** High-density lipoprotein levels in response to fasting: subgroup analysis by fasting group 2 level. **(C)** High-density lipoprotein levels in response to fasting: threshold meta-regression (*k* = 23). **(D)** High-density lipoprotein levels in response to fasting: funnel plot.

Twenty-three independent effect sizes derived from water-only fasting studies were included in the HDL analysis. The random-effects model demonstrated a small but statistically significant reduction in HDL following fasting (g = −0.233, SE = 0.062; 95% CI: −0.355 to −0.111; *p =* 0.0002). Between-study heterogeneity was moderate (Q = 37.42, *p =* 0.021; I^2^ = 41.2%), indicating meaningful variability across study protocols and participant characteristics (Figure 2A).

Robustness analyses supported the stability of this finding. Hartung–Knapp adjusted confidence intervals based on REML τ^2^ produced nearly identical estimates (95% HK CI: −0.348 to −0.116), confirming that the statistical significance of the pooled effect was not sensitive to inference method. The 95% prediction interval ranged from −0.565 to 0.098, indicating that although the average effect suggests a modest HDL reduction, individual future studies may observe smaller or non-significant changes.

Sensitivity analyses confirmed the robustness of this finding. Effect size estimates remained virtually unchanged when alternative pre–post correlation values were applied (r = 0.3: g = −0.234; r = 0.7: g = −0.231). Similarly, restricting the analysis to the longest fasting duration per study yielded a comparable pooled estimate (g = −0.238), indicating that within-study pooling did not materially influence results. Between-study variance estimates were consistent across τ^2^ estimators (DL = 0.0329; PM = 0.0539; REML = 0.0216), supporting the stability of the pooled effect across random-effects specifications. Collectively, these results indicate a modest but robust reduction in HDL concentrations following water-only fasting, with subsequent threshold analyses exploring duration-dependent patterns.

#### 3.3.2 Subgroup analyses by fasting duration

Subgroup analyses were conducted using two duration categories (≤3 days vs. > 3 days), consistent with the threshold-informed classification framework. Short-term fasting (≤3 days; k = 9) was not associated with meaningful changes in HDL concentrations (g = −0.013; 95% CI: −0.230 to 0.204). In contrast, longer fasting durations (>3 days; k = 14) were associated with a significant reduction in HDL (g = −0.325; 95% CI: −0.451 to −0.200). The between-group heterogeneity test was statistically significant (Q < sub > Between</sub > = 7.691, df = 1, *p =* 0.0055), indicating that fasting duration significantly moderated HDL responses. Subgroup forest plots are presented in Figure 2B.

#### 3.3.3 Threshold meta-regression

Threshold meta-regression identified a fasting-duration breakpoint at 3.0 days, indicating a non-linear association between fasting duration and HDL responses. The estimated threshold was supported by bootstrap resampling (95% CI: 2.5 to 11.0 days) and remained stable under leave-one-out diagnostics (range: 2.5 to 11.0 days), suggesting that the breakpoint was not driven by any single study. Prior to the threshold, HDL exhibited a negative slope (*β* = −0.2170), reflecting an early decline in HDL concentrations with increasing fasting duration. Beyond approximately 3 days, the slope became nearly flat (β = −0.0033), indicating attenuation of further decreases and relative stabilization during prolonged fasting. Collectively, these findings reveal an early-phase HDL reduction followed by a plateau pattern, characterized by minimal additional change at longer fasting durations (Table 2). The segmented regression model is illustrated in Figure 2C.

**Table 2:** Threshold meta-regression summary of lipid responses.

Biomarker	Breakpoint τ (days)	Uncertainty interval 95% CI	Slope ≤ τ	Slope > τ	No. of studies within breakpoint region	Interpretation
HDL-C	3.0	2.5–11.0	−0.2170	−0.0033	13	Early decline, plateau
LDL-C	10.0	5.0–11.0	+0.0570	−0.1239	8	Early increase, stabilization or reduction
Total-C	5.0	5.0–11.0	+0.1688	−0.0603	10	Early increase, plateauing or modest reduction
Triglycerides	2.5	1.5–5.0	+0.8889	−0.0468	14	Rapid increase, attenuation and stabilization
VLDL-C	14.0	3.0–16.5	+0.0579	−0.2521	4	Modest increases, tendency toward declining (Interpret cautiously)

τ, breakpoint (threshold) fasting duration; CI, confidence interval; HDL-C, high-density lipoprotein cholesterol; LDL-C, low-density lipoprotein cholesterol; Total-C, total cholesterol; VLDL-C, very-low-density lipoprotein cholesterol.

#### 3.3.4 Publication bias

The funnel plot appeared symmetrical, and Egger’s regression test showed no evidence of small-study effects (intercept = −0.124, SE = 0.758; z = −0.16; *p =* 0.870). Therefore, publication bias is unlikely to meaningfully influence HDL results (Figure 2D).

### 3.4 Low-density lipoprotein (LDL)

#### 3.4.1 Overall meta-analysis

**Figure 3:**
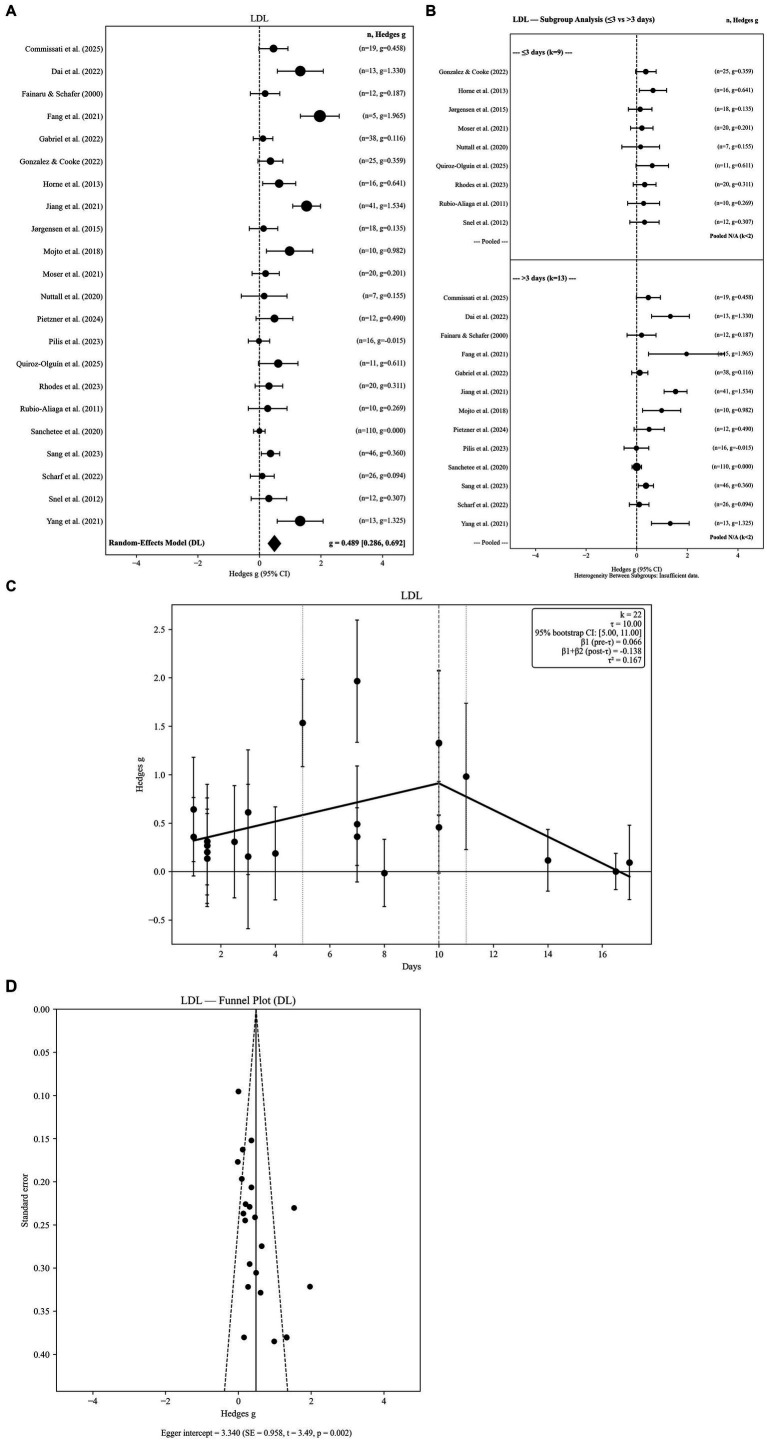
**(A)** Low-density lipoprotein levels in response to fasting. **(B)** Low-density lipoprotein levels in response to fasting: subgroup analysis by fasting group 2 level. **(C)** Low-density lipoprotein levels in response to fasting: threshold meta-regression (*k* = 22). **(D)** Low-density lipoprotein levels in response to fasting: funnel plot.

Twenty-two independent effect sizes were included in the LDL analysis. The random-effects model demonstrated a statistically significant increase in LDL following water-only fasting (g = 0.489, SE = 0.104; 95% CI: 0.286 to 0.692; *p <* 0.001). Between-study heterogeneity was substantial (Q = 92.95, *p <* 0.001; I^2^ = 77.4%), indicating considerable variability across fasting durations, study designs, and participant characteristics (Figure 3A).

Robustness analyses further supported the stability of this finding. Hartung–Knapp adjusted confidence intervals based on REML τ^2^ yielded a comparable effect estimate (95% HK CI: 0.269 to 0.720), confirming that statistical significance was maintained under more conservative inference. However, the 95% prediction interval ranged from −0.472 to 1.450, indicating substantial between-study variability and suggesting that future individual studies may observe attenuated or even non-significant LDL changes despite the positive pooled effect.

Sensitivity analyses confirmed the robustness of this finding. Effect size estimates remained highly consistent across alternative pre–post correlation assumptions (r = 0.3: g = 0.491; r = 0.7: g = 0.482). Restricting analyses to the longest fasting duration per study yielded a comparable pooled effect (g = 0.454), suggesting minimal influence of within-study pooling on the overall estimate. Between-study variance estimates were similar across τ^2^ estimators (DL = 0.1674; PM = 0.2049; REML = 0.2016), supporting the stability of results under different random-effects specifications. Collectively, these results indicate a robust increase in LDL concentrations during water-only fasting, albeit with substantial heterogeneity reflecting duration-dependent and study-specific influences.

#### 3.4.2 Subgroup analyses by fasting duration

Subgroup analyses were performed using two duration categories (≤3 days vs. > 3 days). Short-term fasting (≤3 days; k = 9) was associated with a modest but statistically significant increase in LDL (g = 0.322; 95% CI: 0.151 to 0.494). Longer fasting durations (>3 days; k = 13) demonstrated a larger pooled increase (g = 0.618; 95% CI: 0.304 to 0.932). However, the between-group heterogeneity test was not statistically significant (Q < sub > Between</sub > = 0.000, df = 1, *p =* 0.9852), indicating that categorical fasting duration alone did not explain variability in LDL responses. Subgroup forest plots are presented in Figure 3B.

#### 3.4.3 Threshold meta-regression

Threshold meta-regression identified a fasting-duration breakpoint at 10.0 days, indicating a non-linear association between fasting duration and LDL responses. The estimated threshold was supported by bootstrap resampling (95% CI: 5.0–11.0 days) and remained stable under leave-one-out diagnostics (range: 5.0–11.0 days), suggesting that the breakpoint was not driven by any single study. Prior to the threshold, LDL exhibited a positive slope (*β* = 0.0570), reflecting progressive LDL elevation with increasing fasting duration. Beyond approximately 10 days, the slope reversed (*β* = −0.1239), indicating attenuation and a tendency toward LDL stabilization or reduction during prolonged fasting. Collectively, these findings reveal a biphasic LDL trajectory characterized by early increases followed by plateauing or partial reversal at longer fasting durations (Table 2). The segmented regression model is illustrated in Figure 3C.

#### 3.4.4 Publication bias

Visual inspection of the funnel plot indicated notable asymmetry, with several smaller studies demonstrating disproportionately large effect sizes. Egger’s regression test provided statistical evidence of publication bias (intercept = 3.340, SE = 0.958; z = 3.49; *p =* 0.000). This suggests that available evidence may overestimate the magnitude of LDL increase due to selective publication of studies favoring stronger effects (Figure 3D).

### 3.5 Total cholesterol

#### 3.5.1 Overall meta-analysis

**Figure 4:**
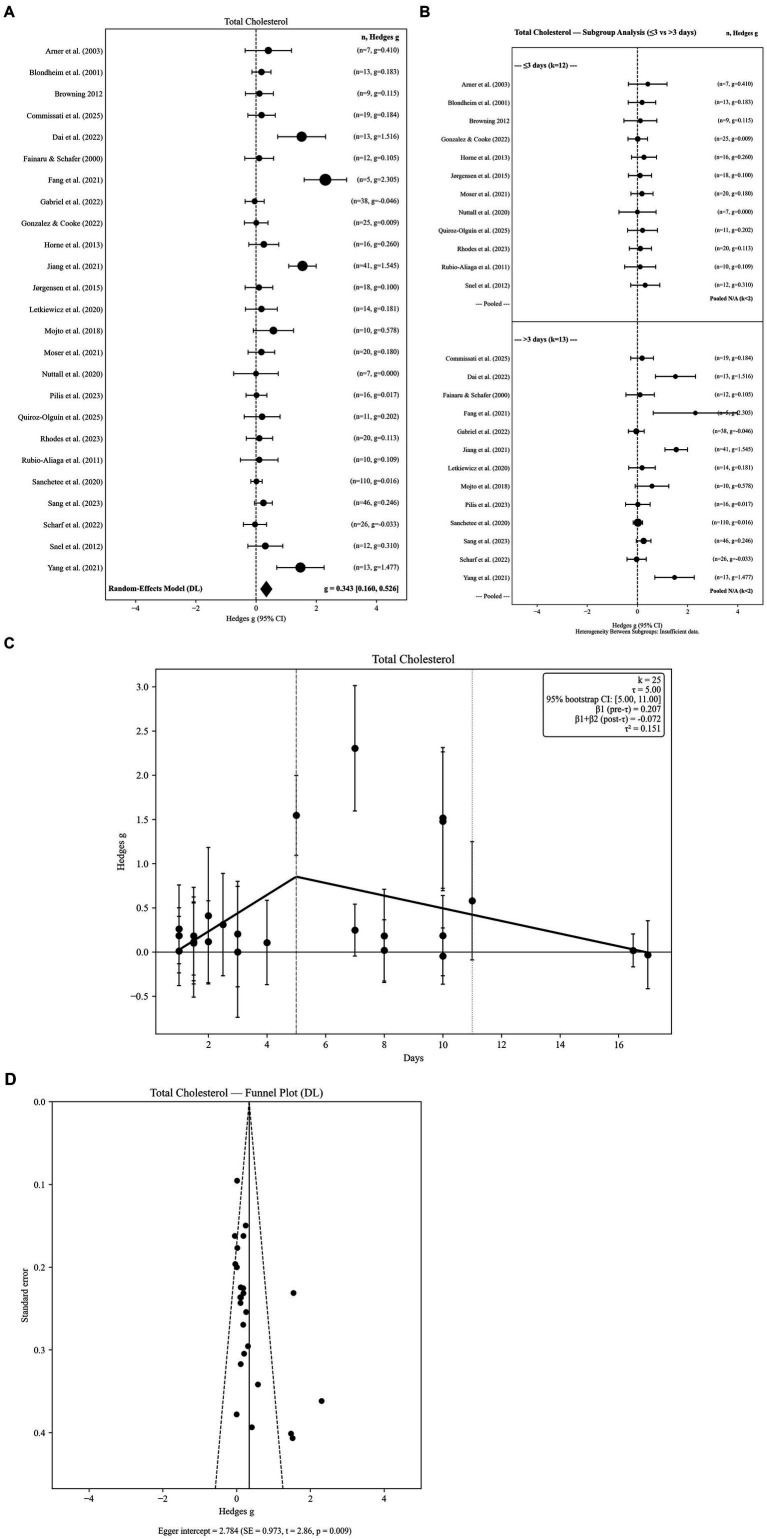
**(A)** Total cholesterol levels in lipoprotein response to fasting. **(B)** Total cholesterol levels in response to fasting-subgroup analysis by fasting group 2 level. **(C)** Total cholesterol levels in response to fasting: Threshold meta-regression (*k* = 25). **(D)** Total cholesterol levels in response to fasting-funnel plot.

Twenty-five independent effect sizes were included in the total cholesterol analysis. The random-effects model demonstrated a statistically significant increase in total cholesterol following water-only fasting (g = 0.343, SE = 0.093; 95% CI: 0.160 to 0.526; *p =* 0.0002). Between-study heterogeneity was substantial (Q = 100.36, *p <* 0.001; I^2^ = 76.1%), indicating considerable variability across fasting durations, participant characteristics, and biochemical assessment methods (Figure 4A).

Robustness analyses further supported the stability of this finding. Hartung–Knapp adjusted confidence intervals based on REML τ^2^ yielded a comparable effect estimate (95% HK CI: 0.136 to 0.578), confirming that statistical significance was preserved under more conservative inference. However, the 95% prediction interval ranged from −0.669 to 1.355, indicating marked between-study variability and suggesting that individual future studies may observe smaller or non-significant changes despite the positive pooled effect.

Sensitivity analyses confirmed the robustness of this effect. Pooled estimates remained consistent under alternative pre–post correlation assumptions (r = 0.3: g = 0.350; r = 0.7: g = 0.330). Restricting analyses to the longest fasting duration per study yielded a comparable effect size (g = 0.312), suggesting minimal influence of within-study pooling. Between-study variance estimates were stable across τ^2^ estimators (DL = 0.1512; PM = 0.2600; REML = 0.2306), supporting the reliability of the pooled result under different random-effects specifications. Overall, these findings indicate a robust elevation in total cholesterol during water-only fasting, albeit with substantial heterogeneity reflecting duration-dependent and study-specific influences.

#### 3.5.2 Subgroup analyses by fasting duration

Subgroup analyses were conducted using two fasting-duration categories (≤3 days vs. > 3 days). Short-term fasting (≤3 days; k = 12) was associated with a small but statistically significant increase in total cholesterol (g = 0.152; 95% CI: 0.013 to 0.291). Longer fasting durations (>3 days; k = 13) demonstrated a larger pooled increase (g = 0.547; 95% CI: 0.222 to 0.871). However, the between-group heterogeneity test was not statistically significant (Q < sub > Between</sub > = 1.324, df = 1, *p =* 0.2499), indicating that categorical fasting duration alone did not significantly explain variability in total cholesterol responses. Subgroup forest plots are presented in Figure 4B.

#### 3.5.3 Threshold meta-regression

Threshold meta-regression identified a fasting-duration breakpoint at 5.0 days, indicating a non-linear association between fasting duration and total cholesterol responses. The estimated threshold was supported by bootstrap resampling (95% CI: 5–11.0 days) and remained stable under leave-one-out diagnostics (range: 5.0–11.0 days), suggesting that the breakpoint was not driven by any single study. Prior to the threshold, total cholesterol exhibited a positive slope (*β* = 0.1688), reflecting progressive elevation during early fasting. Beyond approximately 5 days, the slope reversed (*β* = −0.0603), indicating attenuation and partial stabilization with longer fasting durations. Collectively, these findings suggest a biphasic trajectory of total cholesterol, characterized by early increases followed by plateauing or modest reductions during extended fasting (Table 2). The segmented regression model is illustrated in Figure 4C.

#### 3.5.4 Publication Bias

The funnel plot demonstrated noticeable asymmetry, with several small-sample studies reporting disproportionately large increases. Egger’s regression test indicated evidence of small-study effects (intercept = 2.784, SE = 0.973; z = 2.86; *p =* 0.004), suggesting that publication bias may lead to overestimation of the magnitude of cholesterol increases (Figure 4D).

### 3.6 Triglycerides

#### 3.6.1 Overall meta-analysis

**Figure 5:**
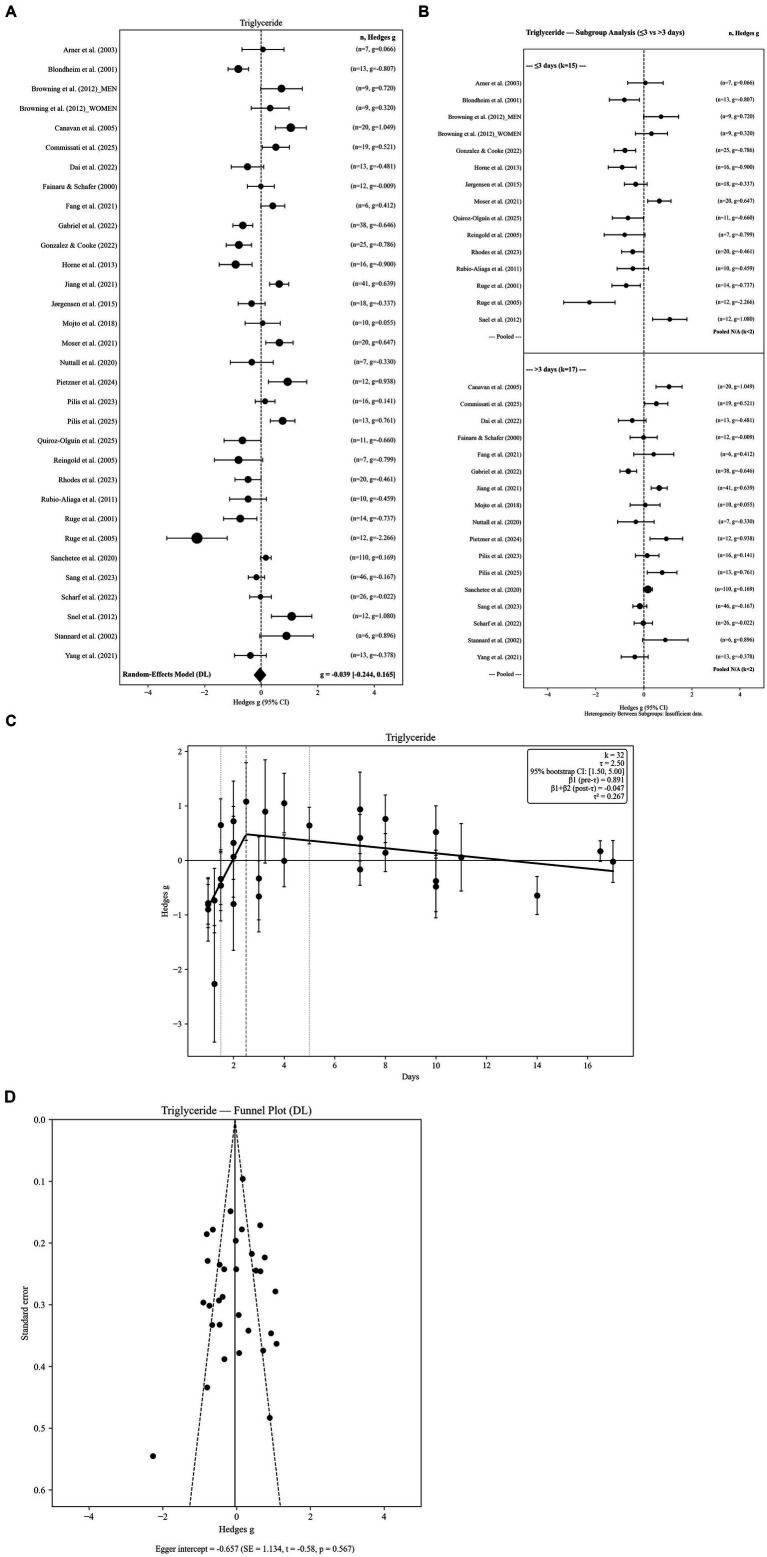
**(A)** Triglycerides levels in response to fasting. **(B)** Triglycerides levels in response to fasting: subgroup analysis by fasting group 2 level. **(C)** Triglycerides levels in response to fasting: threshold meta-regression (*k* = 32). **(D)** Triglycerides levels in response to fasting: funnel plot.

Thirty-two independent effect sizes were included in the triglyceride analysis. The random-effects model indicated no statistically significant overall change in TG concentrations following water-only fasting (g = −0.039, SE = 0.104; 95% CI: −0.244 to 0.165; *p =* 0.7068). Between-study variability was substantial (Q = 180.51, *p <* 0.001), with high heterogeneity observed (I^2^ = 82.8%), reflecting considerable methodological and population-level differences across studies (Figure 5A).

Robustness analyses confirmed the stability of this null finding. Hartung–Knapp adjusted confidence intervals based on REML τ^2^ remained non-significant (95% HK CI: −0.276 to 0.193), indicating that inference was not sensitive to the choice of variance estimator. The 95% prediction interval ranged from −1.273 to 1.194, highlighting pronounced between-study variability and suggesting that individual future studies may report either increases or decreases in triglyceride levels despite the near-zero pooled effect.

Sensitivity analyses demonstrated that this null finding was robust to alternative pre–post correlation assumptions (r = 0.3: g = −0.040; r = 0.7: g = −0.038). Restricting analyses to the longest fasting duration per study produced a similar non-significant estimate (g = −0.077), indicating minimal influence of within-study pooling. Between-study variance estimates were consistent across τ^2^ estimators (DL = 0.2673; PM = 0.3788; REML = 0.3540), supporting the stability of the null effect across random-effects specifications. Overall, these results suggest that water-only fasting does not produce a consistent directional change in triglyceride levels, although substantial heterogeneity persists across studies.

#### 3.6.2 Subgroup analyses by fasting duration

Subgroup analyses were conducted using two fasting-duration categories (≤3 days vs. > 3 days). Short-term fasting (≤3 days; k = 15) showed a trend toward reduced triglyceride levels (g = −0.335; 95% CI: −0.684 to 0.013), although this effect did not reach statistical significance. In contrast, longer fasting durations (>3 days; k = 17) demonstrated a non-significant tendency toward higher TG concentrations (g = 0.187; 95% CI: −0.034 to 0.408). Importantly, the between-group heterogeneity test was statistically significant (Q < sub > Between</sub > = 33.232, df = 1, *p <* 0.001), indicating that fasting duration strongly moderated triglyceride responses despite non-significant pooled effects within each subgroup. Subgroup forest plots are presented in Figure 5B.

#### 3.6.3 Threshold meta-regression

Threshold meta-regression identified a fasting-duration breakpoint at 2.5 days, indicating a non-linear association between fasting duration and triglyceride responses. The estimated threshold was supported by bootstrap resampling (95% CI: 1.5 to 5.0 days) and remained stable under leave-one-out diagnostics (range: 2.0 to 2.5 days), suggesting that the breakpoint was not driven by any single study. Prior to the threshold, triglycerides exhibited a positive slope (*β* = 0.8889), reflecting rapid early changes during the initial fasting phase. Beyond approximately 2.5 days, the slope became slightly negative (*β* = −0.0468), indicating attenuation and stabilization with longer fasting durations. Collectively, these findings suggest that triglyceride responses to water-only fasting are characterized by early dynamic shifts followed by a plateau phase, consistent with the strong subgroup moderation observed between short and longer fasting durations (Table 2). The segmented regression model is illustrated in Figure 5C.

#### 3.6.4 Publication bias

The funnel plot for TG demonstrated a largely symmetric distribution. Egger’s regression test revealed no evidence of small-study effects (intercept = −1.546, SE = 1.087; z = −1.42; *p =* 0.155), suggesting minimal publication bias. These results indicate that TG estimates are relatively robust and unlikely to be inflated by selective reporting (Figure 5D).

### 3.7 Very-low-density lipoprotein

#### 3.7.1 Overall meta-analysis

**Figure 6:**
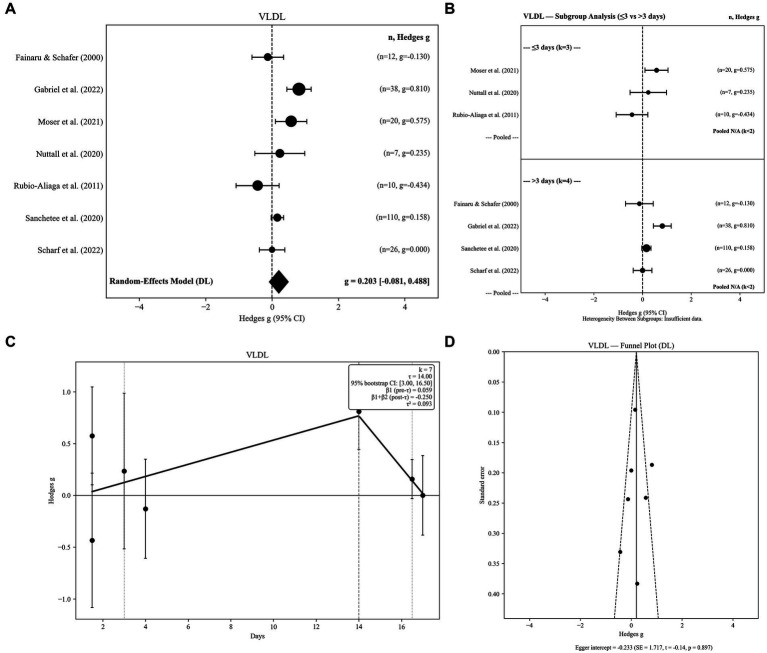
**(A)** Very-low-density lipoprotein levels in response to fasting. **(B)** Very-low-density lipoprotein levels in response to fasting: Subgroup analysis by fasting group 2 level. **(C)** Very-low-density lipoprotein levels in response to fasting: Threshold meta-regression (*k* = 7). **(D)** Very-low-density lipoprotein levels in response to fasting: Funnel plot.

Seven independent effect sizes were included in the VLDL analysis. The random-effects model indicated a small but statistically non-significant increase in VLDL concentrations following water-only fasting (g = 0.203, SE = 0.145; 95% CI: −0.081 to 0.488; *p =* 0.1613). Between-study heterogeneity was substantial (Q = 19.76, *p =* 0.003; I^2^ = 69.6%), indicating meaningful variability across fasting durations, sample characteristics, and analytic procedures (Figure 6A).

Robustness analyses confirmed the stability of this non-significant finding. Hartung–Knapp adjusted confidence intervals based on REML τ^2^ remained non-significant (95% HK CI: −0.150 to 0.551), indicating that statistical inference was not sensitive to the choice of variance estimator. The 95% prediction interval ranged from −0.741 to 1.147, highlighting considerable between-study variability and suggesting that individual future studies may observe either increases or decreases in VLDL despite the near-null pooled effect.

Sensitivity analyses demonstrated that this non-significant finding was robust to alternative pre–post correlation assumptions (r = 0.3: g = 0.213; r = 0.7: g = 0.192). Restricting analyses to the longest fasting duration per study yielded a comparable estimate (g = 0.221), suggesting minimal influence of within-study pooling. Between-study variance estimates were consistent across τ^2^ estimators (DL = 0.0933; PM = 0.1142; REML = 0.1138), supporting stability of the pooled effect across random-effects specifications. Overall, these findings indicate that water-only fasting does not produce a consistent directional change in VLDL concentrations, although moderate-to-high heterogeneity persists across studies.

#### 3.7.2 Subgroup analyses by fasting duration

Subgroup analyses were performed using two fasting-duration categories (≤3 days vs. > 3 days), and findings were interpreted cautiously due to the limited number of available studies. Short-term fasting (≤3 days; k = 3) showed non-significant and highly variable changes in VLDL concentrations (g = 0.150; 95% CI: −0.471 to 0.771). Similarly, longer fasting durations (>3 days; k = 4) demonstrated a small, non-significant increase (g = 0.221; 95% CI: −0.140 to 0.581). The between-group heterogeneity test was not statistically significant (Q < sub > Between</sub > = 0.006, df = 1, *p =* 0.9381), indicating that categorical fasting duration did not meaningfully moderate VLDL responses (Figure 6B).

#### 3.7.3 Threshold meta-regression

Threshold meta-regression identified a fasting-duration breakpoint at 14.0 days, indicating a non-linear association between fasting duration and VLDL responses. The estimated threshold was supported by bootstrap resampling (95% CI: 3.0 to 16.5 days) and remained stable under leave-one-out diagnostics (range: 4.0–14.0 days), suggesting that the breakpoint was not driven by any single study. Prior to the threshold, VLDL exhibited a positive slope (*β* = 0.0579), indicating gradual increases with fasting duration during the early phase. Beyond approximately 14 days, the slope reversed (*β* = −0.2521), suggesting attenuation and a tendency toward declining VLDL concentrations with prolonged fasting. Collectively, these findings suggest a biphasic VLDL trajectory characterized by modest early increases followed by partial reversal at longer fasting durations (Figure 6C). Given the limited number of available VLDL effect sizes (k = 7), these threshold estimates should be interpreted cautiously (Table 2).

#### 3.7.4 Publication bias

The funnel plot showed a largely symmetric pattern of effect sizes. Egger’s regression test indicated no evidence of small-study effects (intercept = −0.233, SE = 1.717; z = −0.14; *p =* 0.892). Overall, VLDL estimates do not appear to be influenced by publication bias (Figure 6D).

A summary of the findings for all lipid parameters is presented in Table 3.

**Table 3:** Changes in lipid parameters during water-only fasting, according to fasting duration.

Lipid parameters	Overall effect (Hedges g, 95% CI, *p* value)	Heterogeneity (I^2^)	≤3 days g (95% CI)	>3 days g (95% CI)	Threshold (days)	Publication bias
HDL-C	g = −0.233, 95% CI: −0.355 to −0.111, *p =* 0.0002	41.2% (moderate)	−0.013 (−0.230, 0.204)	−0.325 (−0.451, −0.200)	3.0*	Not detected (Egger *p =* 0.870)
LDL-C	g = 0.489, 95% CI: 0.286 to 0.692, *p <* 0.001	77.4% (high)	0.322 (0.151, 0.494)	0.618 (0.304, 0.932)	10.0*	Present (Egger *p <* 0.001)
Total Cholesterol	g = 0.343, 95% CI: 0.160 to 0.526, *p =* 0.0002	76.1% (high)	0.152 (0.013, 0.291)	0.547 (0.222, 0.871)	5.0*	Present (Egger *p =* 0.004)
Triglycerides	g = −0.039, 95% CI: −0.244 to 0.165, *p =* 0.7068	82.8% (high)	−0.335 (−0.684, 0.013)	0.187 (−0.034, 0.408)	2.5*	Not detected (Egger *p =* 0.155)
VLDL-C	g = 0.203, 95% CI: −0.081 to 0.488, *p =* 0.1613	69.6% (moderate–high)	0.150 (−0.471, 0.771)	0.221 (−0.140, 0.581)	14.0*	Not detected (Egger *p =* 0.892)

HDL-C: high-density lipoprotein cholesterol; LDL-C: low-density lipoprotein cholesterol; VLDL-C: very-low-density lipoprotein cholesterol; CI: confidence interval; I^2^: heterogeneity statistic; ≤3 days: short-term fasting**; >**3 days: long-term fasting. ^*^Threshold values indicate fasting-duration breakpoints identified by segmented meta-regression. Bold values indicate the identified fasting-duration thresholds.

### 3.8 Risk of bias and certainty of evidence

Risk of bias assessments indicated that most randomized controlled trials were judged as low risk of bias according to RoB 2. Among non-randomized intervention studies assessed using ROBINS-I, the overall risk of bias was predominantly moderate, mainly due to the absence of control groups and potential confounding inherent to single-arm fasting designs. A small number of studies were judged as having serious risk of bias, and no study was classified as critical risk (Supplementary Figures S1, S2).

According to the GRADE framework, certainty of evidence was rated as low for HDL-C and very low for LDL-C, total cholesterol, triglycerides, and VLDL-C. Downgrading was primarily driven by study limitations related to non-randomized designs, substantial heterogeneity across studies, and evidence of small-study effects for LDL-C and total cholesterol. Detailed GRADE assessments are presented in Supplementary Table S2.

## 4 Discussion

This meta-analysis provides a comprehensive synthesis of duration-dependent lipid responses to water-only fasting, revealing a pattern that is both physiologically plausible and more nuanced than previously assumed. Consistent with emerging evidence, our findings show that lipid fractions adapt heterogeneously across fasting du-rations: LDL and total cholesterol exhibit early and progressive increases before reaching a plateau around longer fasts, HDL declines predominantly during multi-day protocols, and triglyceride responses vary across phases of fasting with no uniform overall effect. These results suggest that fasting duration may be an important determinant of lipid dynamics and that short-, and long-term fasts may reflect distinct metabolic states rather than a single, continuous trajectory.

The subgroup forest plot shows that HDL responses varied significantly across studies, particularly in the ≤3-day fasting category (8, 11, 12, 19–21, 25). In the Ru-bio-Aliaga et al. (32) study, HDL remained virtually unchanged after 36 h of fast-ing (from 48.52 ± 7.93 mg/dL to 48.60 ± 9.47 mg/dL), with the smallest effect size (g = 0.000), in this group. In the Arner et al. (13) study, HDL increased from 1.40 ± 0.11 mmol/L to 1.57 ± 0.13 mmol/L, with the largest positive effect size (g = 1.183) in this subgroup. The significant increase in the Arner et al. (13) study is likely related to both the tendency of the very small sample size (*n =* 7) to statistically inflate effect sizes and the measurement within the acute neuroendocrine response window; sympathetic activity, catecholamines, and lipolysis increase sharply during a 48-h fast, which may lead to a transient overshoot in HDL production in some individuals (10, 11). Ru-bio-Aliaga et al.’s (32) study, however, focused on the fasting metabolome rather than the classical lipid profile, performed network analysis across multiple amino and keto acids, FFAs, and ketone bodies, reported HDL as a secondary outcome, and showed a minimal, biologically neutral, statistically neutral change in HDL after 36-h fasting (from 48.52 ± 7.93 mg/dL to 48.60 ± 9.47 mg/dL) (20). Thus, these two extreme studies re-flect different contexts, both in terms of analytical focus, sampling, and timing, and explain a significant portion of the heterogeneity in the ≤3-day subgroup. The overall meta-analysis findings revealed a small but statistically significant decrease in HDL-C after water-only fasting (g = −0.233; 95% CI − 0.355 to −0.111) (Figure 2A). Moderate heterogeneity (I^2^ = 41.2%) suggests that protocol duration, participants’ metabolic status, and timing of measurement are determinants of the HDL response. Subgroup analyses based on fasting duration revealed a clear temporal pattern in HDL-C responses. While no significant change was observed in short-term fasting (≤3 days), fasting longer than 3 days was associated with a significant and consistent de-crease in HDL-C. Significant intergroup heterogeneity confirms that fasting duration is a key determinant of HDL response. Threshold meta-regression further identified a nonlinear relationship with an estimated breakpoint at approximately 3 days. HDL levels decreased in the early phase of fasting but showed minimal further decrease after this point, exhibiting a plateau pattern. This trajectory is consistent with established human fasting physiology, where the transition from glycogen depletion to increased lipolysis and ketogenesis leads to acute metabolic adjustments, followed by stabilization once the body reaches a new metabolic equilibrium. During the first 24–48 h of fasting, hepatic glycogen stores are rapidly depleted, resulting in stimulated lipolysis, increased free fatty acid (FFA) efflux from adipose tissue to the liver, ketone body synthesis initiated, and lipid distribution in the liver and skeletal muscle dramatically altered (14, 23). The decrease in HDL-C during this acute catabolic phase can be explained by the transient suppression of apolipoprotein AI synthesis, faster clearance of HDL-C particles by peripheral tissues, and increased cholesterol and triglyceride ex-change mediated by cholesterol ester transfer protein (CETP) (31, 36, 37). The small and variable direction of HDL-C effects in short-term fasting studies suggests that individual neuroendocrine responses dominate during this early period and that the system has not yet reached a stable catabolic equilibrium (8, 11, 12, 25). The neutrality of the 36-h fasting study by Rubio-Aliaga et al. (32) also supports the fact that HDL-C levels are not yet permanently suppressed in this transition phase. When fasting is ex-tended to 3 days or more, lipolysis and ketogenesis are restored to a more sustainable level; hepatokines such as fibroblast growth factor 21 (FGF-21) are significantly elevated, and fatty acid oxidation and hepatic lipoprotein output are reregulated (38, 39). Fasting studies lasting longer than 3 days report a decrease in HDL-C, suggesting that a common metabolic pattern is dominant rather than individual fluctuations (16–18, 30, 31, 39). Studies in long-term (>7 days) WOF protocols consistently show a decrease in HDL-C; however, intervening dietary histories, medical supervision, and refeeding protocols can contribute to heterogeneity (3–5, 7, 15, 28, 29). The persistently slightly lower HDL level during this phase may be related to prolonged negative energy balance, decreased apolipoprotein synthesis, and a shift in liver synthesis priorities toward gluconeogenesis and ketone production. In this context, the small but significant decrease in HDL-C observed in the meta-analysis can be interpreted as a reflection of this holistic adaptive response to fasting at the lipoprotein level. From a clinical perspective, the magnitude of the HDL decrease observed in this analysis is small and appears to fall below the thresholds typically considered clinically significant in cardiovascular risk assessment (40). Although this decrease theoretically appears negative due to the anti-inflammatory and endothelial-protective properties of HDL-C, the small effect size, the fact that post-refeeding values in many studies are close to baseline, and the lack of evidence of publication bias suggest that the effect of water-only fasting on HDL-C is likely temporary and reversible (3–5, 11, 17). Beyond lipid transport, HDL particles exhibit anti-inflammatory, antioxidant, and endothelial protective functions (40). Fasting-induced lipid remodeling occurs alongside reductions in oxidative stress and inflammatory signaling reported in fasting physiology; these reductions include de-creases in CRP, IL-6, and TNF-*α* in various fasting paradigms (41, 42). In this context, modest decreases in circulating HDL-C concentration do not necessarily imply a decrease in cardioprotective function; functional remodeling of HDL particles and improvements in systemic inflammatory status may offset quantitative decreases. Collectively, these findings support the interpretation that the decrease in HDL during water-only fasting, particularly during fasting periods longer than 3 days, represents an early adaptive metabolic response. While lipid profiles monitoring may be prudent during long-term fasting protocols, the observed changes appear physiologically adaptive, temporally limited, and unlikely to indicate adverse cardiometabolic risk in otherwise healthy individuals.

The overall result of the meta-analysis indicated a significant increase in LDL cholesterol levels after water-only fasting (g = 0.489; 95% CI 0.286–0.692), and high heterogeneity (I^2^ = 77.4%) suggested that this response varied widely depending on protocol duration, study design, and sample characteristics. Forest plots and subgroup analyses showed a modest but statistically significant increase in LDL during short fasting periods (≤3 days) (g = 0.322; 95% CI: 0.151 to 0.494). Fasting periods longer than 3 days showed a larger increase (g = 0.618; 95% CI: 0.304 to 0.932). This pattern clearly demonstrates that the LDL response is time-sensitive and nonlinear, with the more pronounced increase occurring during the >3 fasting period. In the subgroup graphs, the large effects in the long-term fasting time interval are particularly driven by Fang et al. (16) (beego protocol; g = 1.97) and Jiang et al. (17) (5-day WOF; from 3.65 ± 0.96 mmol/L to 5.33 ± 1.16 mmol/L; g = 1.53); both studies reported a significant increase in LDL-C. A common feature of these studies is the strict WOF application, capturing the 4–7 day phase of intense metabolic restructuring, and the accompanying addition-al physiological stresses [in the Fang et al. (16)]. Again in the long-term fasting group, Dai et al. (5) (10 days of fasting; from 3.67 ± 0.85 mmol/L to 5.05 ± 1.05 mmol/L) and Yang et al. (29) (10 days of fasting; from 3.6 ± 1.0 mmol/L to 5.1 ± 1.1 mmol/L) re-ported relatively large increases, while Sanchetee et al. (7) (132.8 ± 37.8 mg/dL be-fore and after fasting) and Pilis et al. (6) (from 146.9 ± 51.6 mg/dL to 148.4 ± 66.4 mg/dL), the near-neutral effects suggest that protocol differences (baseline metabolic status, fluid and electrolyte monitoring, refeeding, and concomitant exercise) significantly modu-late the LDL response. The modest, incremental effects observed in the ≤3-day fasting group in studies such as Horne et al. (11) (increased by 23.1 ± 35.1 mg/dL) and Qui-roz-Olguín et al. (20) (increased from 109.2 mg/dL (95.1–126.8) to 131.0 mg/dL (111.0–160.8) suggest that short-term fasting has the potential to raise LDL but has not yet reached peak levels. Conversely, the near-zero effect in large-sample studies such as Sanchetee et al.’s (7) study supports the possibility of effect inflation in small studies. Threshold meta-regression revealed a turning point in the LDL-C response around day 10; below this time, the LDL curve rises with an increasing slope each day, but after day 10, the slope reverses, and a trend toward stagnation/decrease in LDL is observed (Figure 3C). This biphasic response is consistent with human fasting physiology. In the early-stages of fasting, depletion of liver glycogen stores activates lipolysis in adipose tissue and increases the release of free fatty acids (FFA) into the circulation (10). The increased FFA load is transported to the liver, increasing hepatic lipid flux and stimulating VLDL synthesis (43, 44). A transient increase in LDL-C concentrations may occur as VLDL-C remnants are converted to LDL-C in the circulation (45). These effects of fasting on lipoprotein metabolism have been described as part of the metabolic adaptations observed during fasting (46). In addition, the role of enzymes such as hepatic lipase and cholesterol ester transfer protein (CETP) in lipoprotein remodeling is known, and it has been suggested that these mechanisms may influence LDL-C turnover (47). Indeed, the increase in LDL in short-term (3 ≤ days) WOF trials is compatible with this mechanism (11, 17). During longer fasts, metabolism enters an adaptive phase: hepatokines such as FGF21 increase, hepatic *β*-oxidation intensifies, and the liver’s lipoprotein priorities are recalibrated (38). At this stage, the slope reversal in the threshold me-ta-regression suggests that the effect of prolonged fasting on LDL is no longer “enhancing.” Metabolomic and proteomic data also indicate that energy metab-olism is re-programmed at the molecular level during prolonged fasting, with fatty acid, ketone, and protein networks becoming dominant (32, 39). Therefore, the normalization trend in LDL observed after >10 days can be explained by a shift in liver lipoprotein production to ketogenic metabolism and increased peripheral clearance. However, publication bias was detected in the LDL-C analyses (Figure 3D). The fact that smaller studies more frequently report large effects suggests that the true effect size may have been somewhat overestimated in the meta-analysis (16). Indeed, the more modest LDL change observed in studies with large samples and intensive follow-up (4, 7) supports this interpretation. Clinically, it is important that the increase in LDL becomes particularly pronounced within the >3 days window; lipid monitoring may be considered in individuals with high baseline LDL levels and during long-duration WOF protocols. The tendency for LDL to plat-eau/decrease during prolonged and medically supervised fasting suggests that the potential risk may attenuate over time, but this finding should be interpreted cautiously in light of publication bias.

The overall findings of the meta-analysis indicate a significant increase in total cholesterol levels during water-only fasting (g = 0.343; 95% CI 0.160–0.526), and high heterogeneity (I^2^ = 76.1%) suggests that this response varies widely depending on study duration, participant profile, and protocol characteristics. Figure 4A shows that some studies showed large effect sizes; in particular, Fang et al. (16) (g = 2.31), Jiang et al. (17) (g = 1.55; increased from 5.56 ± 1.03 mmol/L to 7.46 ± 1.36 mmol/L), Dai et al. (5) (g = 1.52; from 4.88 ± 0.76 mmol/L to 6.46 ± 1.16 mmol/L), and Yang et al. (29) (g = 1.48; from 4.9 ± 0.8 mmol/L to 6.5 ± 1.2 mmol/L) reported significant in-creases in cholesterol. In contrast, Nuttall et al. (30) (from 4.0 ± 0.38 mmol/L to 4.0 ± 0.44 mmol/L) and Pilis et al. (6) (from 220.8 ± 60.7 mg/dL to 223.9 ± 71.65 mg/dL) observed near-neutral effects, suggesting that protocol differences such as sample characteristics (metabolic health status), refeeding approaches, and concomi-tant physical activity strongly modulate the cholesterol response. The significantly higher subgroup pooled result (g = 0.547, Figure 4B) in the studies by Jiang et al. (17) and Fang et al. (16), particularly during long-term fasts (>3 days), suggests that this time window is the period of greatest metabolic stress. However, the inclusion of Fang et al. (16) in this group due to their 7-day protocol largely explains this apparent significance. Indeed, when this study is excluded, the effect diminishes, but a high but more stable line will still indicate a significant situation. Subgroup analyses clearly demonstrated that the total cholesterol response was nonlinear and sensitive to time. While a small but reliable increase was observed in fasts lasting ≤3 days (g = 0.152), the largest increase was observed between >3 days (g = 0.547). Threshold me-ta-regression identified a turning point around day 5, below which cholesterol tended to increase, after which the trend reversed and partial normalization began (Figure 4C). This biphasic pattern is consistent with human fasting physiology: early depletion of hepatic glycogen stores accelerates lipolysis, increases free fatty acid (FFA) efflux from adipose tissue to the liver, and elevates liver VLDL production; total cholesterol levels transiently increase during this period. In addition, increased CETP activity and hepatic lipase during fasting may increase the cholesterol pool by accelerating lipo-protein conversion (10, 43, 44, 47). During prolonged fasting, metabolism enters an adaptive phase. Increased hepatokines such as FGF21, enhanced hepatic *β*-oxidation, and altered lipoprotein priorities can lead to a plateau or partial decrease in total cholesterol (38). Metabolomic and proteomic data indicate that energy metabolism is re-programmed at the molecular level during prolonged fasting, with fatty acid and ketone metabolism becoming dominant (32, 39). This physiological restructuring explains the slope reversal detected after >5 days in the threshold meta-regression. However, evidence of publication bias (Figure 4D) suggests that small studies more frequently report large effects; therefore, the magnitude of the large increases during long-term fasting should be interpreted with caution in clinical practice. From a clinical perspective, the most pronounced increase in total cholesterol occurs >3 days, highlighting the potential value of lipid monitoring during this period, particularly in individuals with high baseline cholesterol levels. In long-term, medically supervised WOF protocols, the tendency for cholesterol to plateau/normalize with adaptation suggests that the risk may stabilize over time. However, individualized assessment may be warranted due to publication bias and high heterogeneity. In conclusion, total cholesterol exhibits a duration-dependent response to water-only fasting, increasing significantly in both short- and longer-duration fasts, with a greater elevation beyond 3 days. A threshold around day 5 suggests a subsequent decline, potentially reflecting metabolic adaptation during prolonged fasting. Increases in LDL and total cholesterol during short periods of fast-ing may reflect increased mobilization of cholesterol from peripheral tissues and he-patic lipoprotein remodeling rather than an atherogenic shift. During fasting, increased lipolysis and hepatic lipid influx temporarily increase circulating cholesterol; however, fasting is also associated with a decrease in oxidative stress and systemic inflammatory mediators [including C-reactive protein (CRP), interleukin-6 (IL-6), and tumor necrosis factor-*α* (TNF-α)], which may mitigate the atherogenic potential of these lipid changes (41, 42).

This meta-analysis demonstrates that the effect of water-only fasting on triglyceride (TG) levels does not produce a fixed-direction response, but rather exhibits a bi-phasic pattern significantly influenced by duration. The overall effect analysis revealed a small, statistically insignificant decrease in triglycerides (g = −0.039), but the extremely high heterogeneity (I^2^ = 82.8%) suggests that interindividual metabolic responses and interprotocol differences strongly modulate the TG response (Figure 5A). This suggests that triglyceride metabolism is driven by distinct physiological mechanisms, ranging from short-term energy regulation to long-term adaptation. The wide distribution of effect sizes across individual studies in Figure 5A clearly reflects this heterogeneity. For example, the large effect size reported in Ruge et al. (27) (g = −2.266; from 1.5 ± 0.2 mmol/L to 1.0 ± 0.1 mmol/L) may be attributable to a dramatic in-crease in lipoprotein lipase (LPL) activity during short-term fasting and the rapid clearance of circulating triglycerides to peripheral tissues. In contrast, the TG increases (g = 0.9) observed in Stannard et al. (23) (from 0.76 ± 0.08 mmol/L to 1.16 ± 0.19) and Pietzner et al. (39) (from 0.81 ± 0.37 mM to 1.15 ± 0.29 mM) may be related to increased hepatic VLDL production and accelerated free fatty acid (FFA) reesterifi-cation during the long-term fasting (>3 days). On the other hand, the reporting of minimal changes in studies such as Arner et al. (13) (from 0.69 ± 0.10 mmol/L to 0.71 ± 0.09 mmol/L) and Mojto et al. (28) (from 1.086 ± 0.17 mmol/L to 1.095 ± 0.09 mmol/L) suggests that individual differences in metabolic flexibility, initial lipid profile, and hormonal adaptation are decisive. Subgroup analyses more clearly demonstrated the relationship between this physiological variation and duration. The decrease in TGs observed during ≤3 days of fasting (g = −0.335) can be explained by the rapid decline in insulin levels in the early fasting phase, which increases hormone-sensitive lipase (HSL) activation and accelerates peripheral utilization of plasma TGs (25). During this phase, tissues rapidly utilize FFA, and TG clearance via lipoprotein lipase increases (27). In contrast, the increase in TGs (g = 0.187) in the fasting group (longer than 3 days) indicates increased VLDL resynthesis in the liver in response to a significant increase in hepatic fatty acid influx during the advanced phase of the catabolic process. Prolonged lipolysis and the FFA load directed to the liver, in particular, may trigger a transient increase in TG synthesis (23, 39). The increase in lipid mobilization following leptin suppression demonstrated by Canavan et al. (22) also provides a mechanism supporting the TG elevations occurring in this phase (from 74 ± 7 mg/dL to 114 ± 9 mg/dL) (22). The increase in triglyceride levels neutralizes again during longer periods of fasting (e.g., fasting longer than 7 days), indicating that metabolic adaptation has entered a more balanced phase. During this period, ketone bodies become the predominant energy substrate, reducing the need for hepatic TG synthesis and increasing the direct oxidation of fatty acids by peripheral tissues (14, 39). Indeed, as shown by Gabriel et al. (4) and Dai et al. (5) (from 1.90 ± 1.08 mmol/L to 1.49 ± 0.30 mmol/L), the stabilization of TGs after long-term fasting is a reflection of metabolic reprogramming. Threshold meta-regression results indicate that the sharpest change in triglyceride re-sponse occurs within the 2.5 days. In fasts shorter than 3 days, triglyceride levels tended to decrease slightly, although this change was not statistically significant. In contrast, fasting durations of ≥3 days were associated with a modest, non-significant increase in triglycerides. This directional shift likely reflects the metabolic transition from glycogen depletion to enhanced lipolysis and increased free fatty acid flux. As metabolic adaptation progresses, triglyceride dynamics appear to stabilize, suggesting a regulated balance between lipid mobilization and utilization during prolonged fasting (23, 25). Therefore, assessments of TG should be interpreted not only based on the presence of starvation but also on its duration, and clinical interpretation should consider this dynamic variability.

In this meta-analysis, the effect of water-only fasting on VLDL-C showed a positive trend in direction although it was not statistically significant (g = 0.203). However, the high heterogeneity between studies (I^2^ = 69.6%) suggests that individual physiological responses differ significantly. The most notable increase among studies was seen in the study of Gabriel et al. (4) (g = 0.810) (from 0.49 mmol/L (IQR: 0.44–0.64) to 0.60 mmol/L (IQR: 0.54–0.69), whereas the smallest effect size was reported in the study of Scharf et al. (3) (g = 0.000) (from 25.7 ± 10.3 mg/dL to 25.5 ± 4.5 mg/dL). The increase in VLDL-C in the study of Gabriel et al. (4) can be explained by increased hepatic triglyceride production and accelerated cycling of free fatty acids to the liver after prolonged fasting. Increased lipolysis during prolonged fasting increases circulating FFA levels, and these fatty acids can be re-esterified in the liver and increase VLDL-C synthesis (14, 25). In contrast, in the study of Scharf et al. (3), a significant inflammatory response and hepatic stress were observed during fasting in clinically stabilized participants under close follow-up; It is thought that this situation may have led to the suppression of apolipoprotein B production and thus the limitation of VLDL secretion (3, 39). In time-dependent analyses, the VLDL-C response was found to be heterogeneous and statistically insignificant in short-term fasting (≤3 days), while an increasing trend was evident in the long-term fasting (>3 days) group, but this did not reach statistical significance. However, the breakpoint identified around day 14 in the threshold me-ta-regression indicates that VLDL-C metabolism exhibits a two-phase adaptation. In the first phase, increased lipolysis and FFA flux increase hepatic VLDL-C synthesis. However, during prolonged fasting, VLDL-C production tends to decline again due to hepatic substrate depletion, the dominance of mitochondrial *β*-oxidation, and the use of ketone bodies as the primary energy substrate. This physiological transition explains the increased triglyceride production in the liver during the early phase of fasting and the suppression of lipoprotein synthesis in the later phase, accompanied by the shift to energy economy (14, 38). The decrease in VLDL-C levels observed in Rubio-Aliaga et al.’s (32) short-term (36-h) fasting protocol reflects this early phase of the metabolic transition. This study examined the metabolomic response to fasting and reported that ketone bodies began to predominate over VLDL-C. Similarly, in a study by Nuttall et al. (30) involving diabetic individuals, the limited increase in VLDL-C was associated with increased VLDL-C clearance due to insulin deficiency and suppressed hepatic production. In conclusion, VLDL-C levels do not show a homogeneous response during water-only fasting; they may initially increase depending on the duration of fasting, then become suppressed with metabolic adaptation. Therefore, VLDL may be considered a dynamic biomarker related to hepatic energy balance, not a static parameter in fasting physiology. Triglyceride and VLDL dynamics are related to inflammatory pathways. Increased free fatty acid influx during short-term fasting can temporarily affect hepatic VLDL secretion, while prolonged fasting and ketone use are associated with increased insulin sensitivity and decreased systemic inflammation (48). These adaptations may contribute to a metabolic environment characterized by a reduced inflammatory load despite short-term lipid fluctuations.

These findings suggest that lipid responses to water-only fasting vary by duration and may reflect adaptive metabolic transitions rather than pathological changes. Given the low to very low certainty of the available evidence, the identified duration-related thresholds should be interpreted cautiously and viewed as hypothesis-generating indicators of metabolic adaptation.

### 4.1 Strengths and limitations

A major strength of the present study is the comprehensive evaluation of lipid-specific and duration-dependent responses to water-only fasting using both sub-group analyses and threshold meta-regression, enabling identification of potential non-linear effects that are often obscured in conventional meta-analyses. The inclusion of a broad range of fasting durations and multiple lipid fractions further enhances the clinical relevance of the findings. Nevertheless, our study has several limitations that should be considered when interpreting the findings. Despite these strengths, some limitations must also be considered. Significant heterogeneity existed among the studies in terms of fasting duration, participant characteristics, and biochemical assess-ment methods, which may have affected the combined estimates. Although measurements taken at the end of the fasting period were included in the analysis for post-measurements, minor differences in sampling schedules may have contributed to inter-study heterogeneity. While publication bias was not evident for all outcomes, statistical evidence of small study effects for LDL and total cholesterol suggests that effect sizes may have been overestimated. Many of the included studies relied on pre-post designs without parallel control groups, which limited causal inference. The overall low and very low certainty ratings of the evidence limit the strength of inference and preclude direct clinical recommendations. Additionally, significant variability in refeeding protocols and limited reporting of post-refeeding lipid measurements hindered formal assessment of recovery dynamics and their impact on lipid responses. Furthermore, underreporting of baseline metabolic status, medication use, and dietary history restricted the evaluation of potential effect modifiers. Randomized controlled trials with standardized fasting and refeeding protocols, comprehensive metabolic profiling, and long-term follow-up are needed in the future.

## 5 Conclusion

Water-only fasting appears to produce fraction-specific and time-dependent lipid responses characterized by early metabolic changes followed by adaptive stabilization. HDL concentrations show a modest early decrease that stabilizes after approximately 3 days, whereas LDL and total cholesterol tend to increase during the early stages of fasting and may attenuate or partially reverse with prolonged fasting. Trigliseride responses are heterogeneous, with slight reductions observed during shorter fasting periods and modest increases during longer durations, while VLDL changes remain inconsistent and should be interpreted cautiously given the limited evidence. Overall, the modest magnitude and potentially transient nature of these changes suggest limited clinical significance in healthy individuals. In addition, given the low to very low certainty of the available evidence, the identified duration-related patterns should be interpreted cautiously and viewed as hypothesis-generating indicators of metabolic adaptation rather than clinically actionable thresholds. Further well-designed prospective and controlled studies are needed to confirm these observations and clarify their clinical relevance.

## Data Availability

The original contributions presented in the study are included in the article/Supplementary material, further inquiries can be directed to the corresponding authors.

## Supplementary material

Supplementary Figure S1 Risk of bias summary for randomized controlled trials assessed using the RoB 2 tool.

Supplementary Figure S2 Risk of bias summary for non-randomized intervention studies assessed using the ROBINS-I tool.
