# Supplementary material for: Duration-dependent effects of water-only fasting on blood lipids: a systematic review, meta-analysis, and threshold meta-regression
Source: Front Nutr. 2026 Apr 1;13:1772246. doi: 10.3389/fnut.2026.1772246 (PMC13079636; doi:10.3389/fnut.2026.1772246)
Supplement: Supplementary Figure S1 — Risk of bias summary for randomized controlled trials assessed using the RoB 2 tool. [file Supplementary_File_1.zip › Supplementary Materials/Supplementary Table S1.docx]

Supplementary Material

# Supplementary Table and Figures

## Supplementary Table

**Supplementary Table S1.** Full Search Strategies for Each Database

| **Database** | **Search String (Boolean Query)** |
| --- | --- |
| **PubMed** | (“water only fast”[tiab] OR “water-only fast”[tiab] OR “water only fasting”[tiab] OR “water-only fasting”[tiab] OR “complete fasting”[tiab] OR “total fasting”[tiab] OR “prolonged fasting”[tiab] OR “zero calorie fasting”[tiab] OR “zero-calorie fasting”[tiab] OR “absolute fasting”[tiab] OR “dry fasting”[tiab] OR “72 hour fast”[tiab] OR “36 hour fast”[tiab] OR “multi-day fast”[tiab] OR “food deprivation”[tiab] OR “calorie deprivation”[tiab] OR “Yom Kippur”[tiab] OR “religious fast”[tiab]) AND (“lipid profile”[tiab] OR “serum lipids”[tiab] OR “plasma lipids”[tiab] OR “triglycerides”[tiab] OR “triacylglycerol”[tiab] OR “TG”[tiab] OR “total cholesterol”[tiab] OR “HDL cholesterol”[tiab] OR “LDL cholesterol”[tiab] OR “VLDL”[tiab] OR “non-HDL”[tiab] OR “lipoprotein”[tiab]) AND (humans[MeSH Terms] OR humans[tiab] OR adult[tiab] OR adults[tiab]) |
| **Scopus** | (TITLE-ABS-KEY(“water only fast” OR “water-only fasting” OR “complete fasting” OR “zero calorie fasting” OR “absolute fasting” OR “dry fasting” OR “multi day fasting” OR “prolonged fasting” OR “72 h fast” OR “36 h fast”)) AND (TITLE-ABS-KEY(“lipid profile” OR “triglycerides” OR “total cholesterol” OR “LDL cholesterol” OR “HDL cholesterol” OR “VLDL” OR “non-HDL”)) AND (TITLE-ABS-KEY(human OR participant OR adult)) |
| **Web of Science** | TS=(“water-only fasting” OR “water only fasting” OR “complete fasting” OR “total fasting” OR “zero-calorie fasting” OR “absolute fasting” OR “dry fasting” OR “prolonged fasting” OR “72 hour fast” OR “36 hour fast”) AND TS=(“lipid profile” OR “serum lipids” OR “triglycerides” OR “total cholesterol” OR “HDL cholesterol” OR “LDL cholesterol” OR “lipoprotein”) AND TS=(human OR adults OR participants OR volunteers) |

**Notes:**

1. The search was limited to peer-reviewed articles in English involving human participants.
2. No filters for study design or fasting duration were applied during the initial retrieval stage.
3. The same set of keywords and Boolean operators was adapted to match each database’s indexing system.
4. Reference lists of included studies were also manually checked to identify any additional eligible papers.
