# Supplementary material for: Duration-dependent effects of water-only fasting on blood lipids: a systematic review, meta-analysis, and threshold meta-regression
Source: Front Nutr. 2026 Apr 1;13:1772246. doi: 10.3389/fnut.2026.1772246 (PMC13079636; doi:10.3389/fnut.2026.1772246)
Supplement: Supplementary Figure S1 — Risk of bias summary for randomized controlled trials assessed using the RoB 2 tool. [file Supplementary_File_1.zip › Supplementary Materials/Supplementary Table S2.docx]

**Supplementary Table S2.** GRADE certainty of evidence (outcome-level) for lipid outcomes.

| **Outcome** | **Studies (RCT/NRS)** | **Participants (N)** | **Effect size (Hedges g, 95% CI)** | **Heterogeneity (I²)** | **Publication bias (Egger p)** | **Certainty of evidence (GRADE)** | **Reasons for downgrading** |
| --- | --- | --- | --- | --- | --- | --- | --- |
| **HDL-C** | 23 (4 RCT, 19 NRS) | 509 | −0.233 (−0.355 to −0.111) | 41.2% | 0.870 | **Low** | Study limitations (predominance of non-randomized designs) |
| **LDL-C** | 22 (4 RCT, 18 NRS) | 500 | 0.489 (0.286 to 0.692) | 77.4% | <0.001 | **Very low** | Study limitations; substantial heterogeneity; small-study effects |
| **Total cholesterol** | 25 (4 RCT, 21 NRS) | 531 | 0.343 (0.160 to 0.526) | 76.1% | 0.004 | **Very low** | Study limitations; substantial heterogeneity; small-study effects |
| **Triglycerides** | 31 (5 RCT, 26 NRS) | 611 | −0.039 (−0.244 to 0.165) | 82.8% | 0.155 | **Very low** | Study limitations; very high heterogeneity; imprecision (CI crosses null) |
| **VLDL-C** | 7 (1 RCT, 6 NRS) | 223 | 0.203 (−0.081 to 0.488) | 69.6% | 0.892 | **Very low** | Study limitations; heterogeneity; imprecision (few studies) |

Abbreviations: RCT, randomized controlled trial; NRS, non-randomized study; HDL-C, high-density lipoprotein cholesterol; LDL-C, low-density lipoprotein cholesterol; VLDL-C, very-low-density lipoprotein cholesterol; CI, confidence interval; I², inconsistency statistic; GRADE, Grading of Recommendations Assessment, Development and Evaluation.
