# Supplementary figures and images for: Duration-dependent effects of water-only fasting on blood lipids: a systematic review, meta-analysis, and threshold meta-regression
Source: Front Nutr. 2026 Apr 1;13:1772246. doi: 10.3389/fnut.2026.1772246 (PMC13079636; doi:10.3389/fnut.2026.1772246)

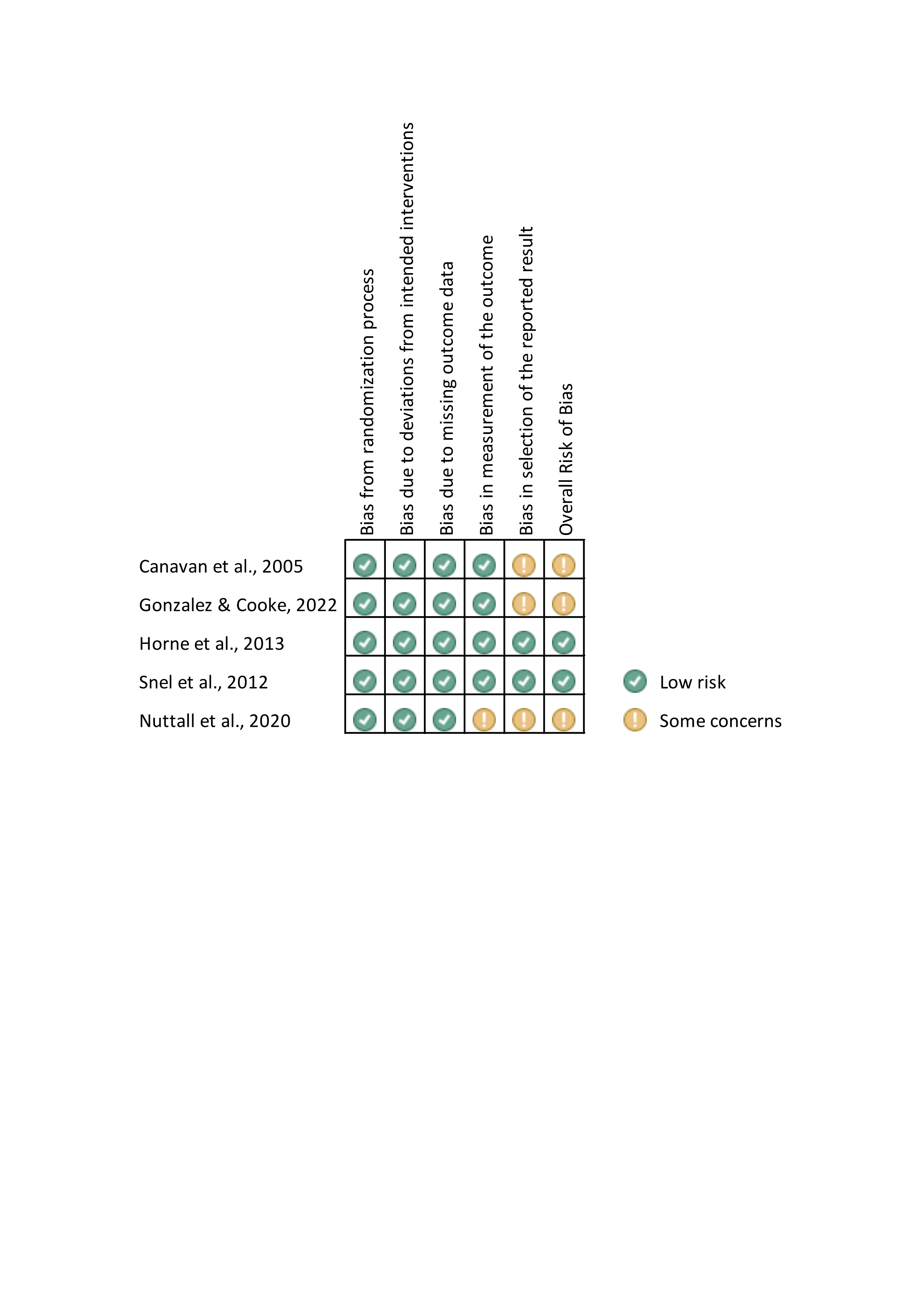

Supplement: Supplementary Figure S1 — Risk of bias summary for randomized controlled trials assessed using the RoB 2 tool. [file Supplementary_File_1.zip › Supplementary Materials/Supplementary Figure S1.png]

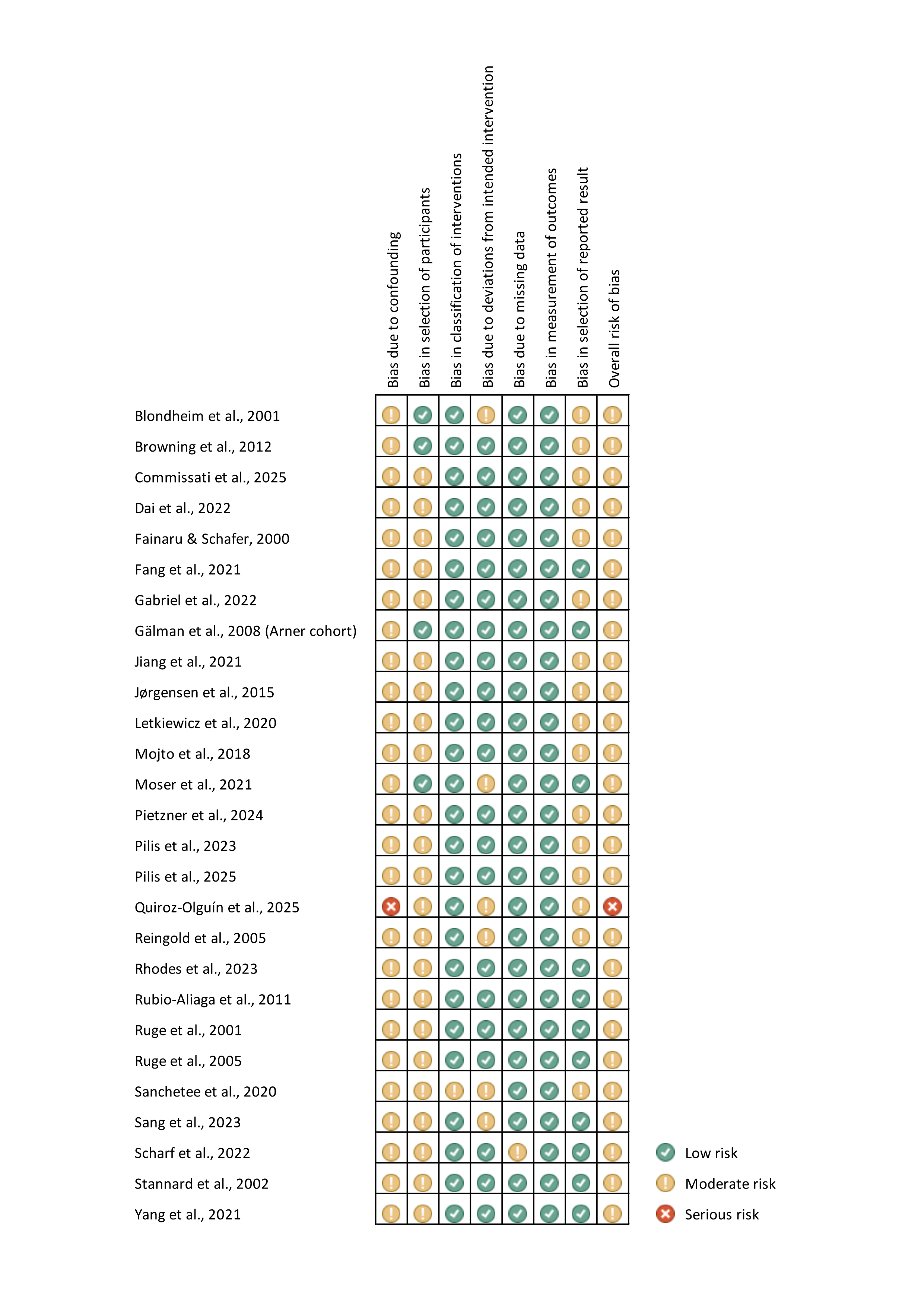

Supplement: Supplementary Figure S1 — Risk of bias summary for randomized controlled trials assessed using the RoB 2 tool. [file Supplementary_File_1.zip › Supplementary Materials/Supplementary Figure S2.png]
